# Surface translocation of ACE2 and TMPRSS2 upon TLR4/7/8 activation is required for SARS-CoV-2 infection in circulating monocytes

**DOI:** 10.1038/s41421-022-00453-8

**Published:** 2022-09-09

**Authors:** Yi Yao, Kalpana Subedi, Tingting Liu, Namir Khalasawi, Carla Diana Pretto-Kernahan, Jesse William Wotring, Jie Wang, Congcong Yin, Aimin Jiang, Chunmei Fu, Peter Dimitrion, Jia Li, Jesse Veenstra, Qijun Yi, Kathy McKinnon, John Ernest McKinnon, Jonathan Zachary Sexton, Li Zhou, Qing-Sheng Mi

**Affiliations:** 1 Center for Cutaneous Biology and Immunology Research, Department of Dermatology, Henry Ford Health System, Detroit, MI USA; 2 Immunology Research Program, Henry Ford Cancer Institute, Henry Ford Health System, Detroit, MI USA; 3 Internal Medicine, Division of Gastroenterology and Hepatology, University of Michigan, Ann Arbor, MI USA; 4 University of Michigan Center for Drug Repurposing, University of Michigan, Ann Arbor, MI USA; 5 Department of Medicinal Chemistry, College of Pharmacy, University of Michigan, Ann Arbor, MI USA; 6 Department of Biochemistry, Microbiology, and Immunology, School of Medicine, Wayne State University, Detroit, MI USA; 7 Department of Public Health Sciences, Henry Ford Health System, Detroit, MI USA; 8 Division of Rheumatology, Department of Internal Medicine, Henry Ford Health System, Detroit, MI USA; 9 Division of Infectious Diseases, Department of Internal Medicine, Henry Ford Health System, Detroit, MI USA; 10 Department of Internal Medicine, Henry Ford Health System, Detroit, MI USA

**Keywords:** Innate immunity, Mechanisms of disease

## Abstract

Infection of human peripheral blood cells by SARS-CoV-2 has been debated because immune cells lack mRNA expression of both angiotensin-converting enzyme 2 (ACE2) and transmembrane serine protease type 2 (TMPRSS2). Herein we demonstrate that resting primary monocytes harbor abundant cytoplasmic ACE2 and TMPRSS2 protein and that circulating exosomes contain significant ACE2 protein. Upon ex vivo TLR4/7/8 stimulation, cytoplasmic ACE2 was quickly translocated to the monocyte cell surface independently of ACE2 transcription, while TMPRSS2 surface translocation occurred in conjunction with elevated mRNA expression. The rapid translocation of ACE2 to the monocyte cell surface was blocked by the endosomal trafficking inhibitor endosidin 2, suggesting that endosomal ACE2 could be derived from circulating ACE2-containing exosomes. TLR-stimulated monocytes concurrently expressing ACE2 and TMPRSS2 on the cell surface were efficiently infected by SARS-CoV-2, which was significantly mitigated by remdesivir, TMPRSS2 inhibitor camostat, and anti-ACE2 antibody. Mass cytometry showed that ACE2 surface translocation in peripheral myeloid cells from patients with severe COVID-19 correlated with its hyperactivation and PD-L1 expression. Collectively, TLR4/7/8-induced ACE2 translocation with TMPRSS2 expression makes circulating monocytes permissive to SARS-CoV-2 infection.

## Introduction

Angiotensin-converting enzyme 2 (ACE2) and accessory protease transmembrane serine protease 2 (TMPRSS2) are needed for severe acute respiratory syndrome coronavirus 2 (SARS-CoV-2) cellular entry, and their expression sheds light on viral tropism and the impact of viral infection throughout the body^1^. SARS-CoV-2 binds to human ACE2 through the viral spike (S) protein, while TMPRSS2 can provide proteolytic cleavage of the S protein for cellular entry^2^. Expression of ACE2 and TMPRSS2 has been described in several cell types, such as endothelial progenitor cells, alveolar epithelial cells, enterocytes of the small intestine, and arterial smooth muscle cells^3–5^. SARS-CoV-2 viral components (RNA and proteins) have been identified in multiple organs, including lungs, heart, intestines, brain, and kidneys, and in various body fluids such as mucus, saliva, urine, serum, and cerebrospinal fluid, suggesting systemic SARS-CoV-2 infection and viremia in COVID-19 patients^6–9^. However, given the lack of ACE2 and TMPRSS2 mRNA expression in blood immune cells, whether circulating immune cells can be infected by SARS-CoV-2 and if infected immune cells contribute to the SARS-CoV-2 systemic distribution are currently under debating.

To date, accumulated single-cell RNA sequencing (scRNA-seq) studies from human tissues have shown that ACE2 and TMPRSS2 mRNA expressions are scarcely detected in most tissue-resident macrophages and peripheral immune cells from healthy individuals^1,10,11^. Based on this, few studies have investigated blood monocyte infection by SARS-CoV-2. By measuring viral load with RT-PCR, Rodrigues et al. reported that SARS-CoV-2 can infect human monocytes in vitro^12^. Grant et al. showed that SARS-CoV-2 transcripts are present in both tissue-resident and monocyte-derived alveolar macrophages from patients with severe COVID-19, and that the virus appears to actively replicate in infected alveolar macrophages^13^. Most recently, Junqueira et al. found that 6% of blood monocytes in COVID-19 patients are infected with SARS-CoV-2 by Fcγ receptor (CD16/CD64)-mediated uptake^14^. However, this finding does not rule out the possible role of ACE2 surface expression in the infection of monocytes or macrophages, which is supported by a recent study showing that human ACE2^+^ macrophages from humanized mice were permissive to SARS-CoV-2 infection mediated by both ACE2 and the Fcγ receptor CD16^15^. The reasons for the discrepancy between ACE2 cell surface protein expression and mRNA expression in SARS-CoV-2 infected monocytes and macrophages remain unclear.

Here we show that circulating resting CD14^+^ monocytes express abundant cytoplasmic, but not surface, ACE2 and TMPRSS2 proteins. Upon ex vivo Toll-like receptor (TLR) 4/7/8 stimulation, intracellular ACE2 translocates to the cell surface independent of ACE2 transcription, and this translocation is blocked by inhibition of endosomal trafficking, suggesting that ACE2 trafficking to the cell surface occurs via exocytosis, while TMPRSS2 surface translocation occurs with a concomitant increase in TMPRSS2 transcription. Monocytes that have both ACE2 and TMPRSS2 protein at the cell surface were efficiently infected by SARS-CoV-2, which was significantly mitigated by inhibiting viral replication with remdesivir, inhibiting TMPRSS2 with camostat, and neutralizing ACE2 with an anti-ACE2 antibody. Furthermore, ACE2 surface translocation in peripheral myeloid cells from patients with COVID-19 correlated with its hyperactivation status.

## Results

### ACE2 is expressed on the cell surface of circulating immune cells upon ex vivo TLR stimulation

**Fig. 1:**
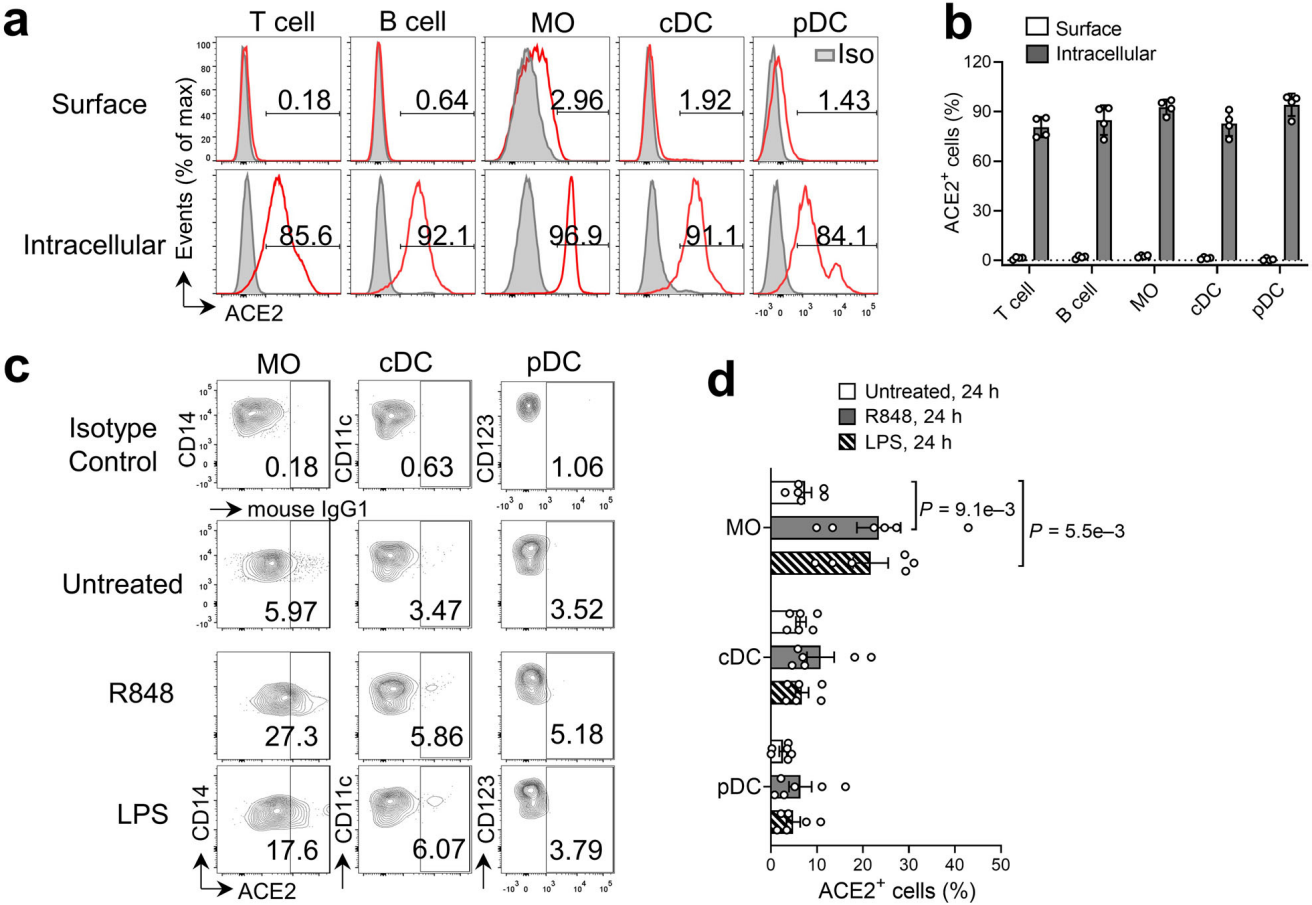
Upregulation of ACE2 expression on the cell surface of peripheral blood myeloid populations upon ex vivo TLR stimulation. **a** Representative flow cytometry analysis of ACE2 protein expression (red line) in T cells, B cells, CD14^+^ MO, cDCs, and pDCs from fresh PBMCs isolated from healthy donors. Mouse IgG1 antibody (gray tinted) was used as isotype control. **b** Frequency of cells expressing cell surface and cytoplasmic ACE2 as in (**a**) (*n* = 4 biologically independent samples/group). **c** PBMCs were cultured ex vivo with or without R484 or LPS for 24 h, followed by flow cytometry analysis of surface ACE2 in CD14^+^ MO, cDCs, and pDCs. Mouse IgG1 antibody was used as the isotype control. **d** Frequencies of indicated cell types expressing surface ACE2 as in **c** (*n* = 6 biologically independent samples/group). cDC classical dendritic cells, pDC plasmacytoid DCs, MO monocytes, LPS lipopolysaccharide.

To determine whether human circulating immune cells express ACE2 protein under resting conditions, we used flow cytometry to examine the surface expression of ACE2 protein in the principal immune cell populations, including T cells, B cells, CD14^+^ monocytes, classical dendritic cells (cDC), and plasmacytoid DCs (pDC) in fresh peripheral blood mononuclear cells (PBMC) from healthy donors (Supplementary Fig. S1). Consistent with the study by Junqueira et al.^14^, both circulating lymphoid and myeloid populations had negligible ACE2 protein on the cell surface (Fig. 1a, b). As expected, sorted CD14^+^ monocytes and CD3^+^ T cells from resting PBMC had nominal expression of ACE2 mRNA that was 10 to 100-fold lower than control cells (Supplementary Fig. S2a, b). Surprisingly, ACE2 intracellular staining showed that the majority (>70%) of circulating immune cell populations, including T cells, B cells, monocytes, and DCs contained abundant ACE2 protein in the cytoplasm (Fig. 1a, b).

TLR7/8 detects single-stranded RNA (such as the SARS-CoV-2 genome), while TLR4 can be activated directly by viral proteins, including SARS-CoV-2 spike proteins^16^, or indirectly by danger signals triggered by viral infection^17,18^. To determine whether ACE2 surface expression could be induced in PBMCs upon TLR stimulation, we treated PBMCs ex vivo with the TLR7/8 ligand resiquimod (R848) or the TLR4 ligand lipopolysaccharide (LPS, *E.coli* serotype O111:B4) for 24 h. We found that the surface-localized ACE2 was markedly increased in CD14^+^ monocytes after treatment with either ligand, while marginal surface ACE2 expression was found in cDC and pDC following TLR stimulation (Fig. 1c, d). We also observed a robust surface ACE2 expression in total T cells, CD4^+^ T cells, and CD8^+^ T cells after ex vivo stimulation with phorbol myristate acetate (PMA)/ionomycin (P/I) (Supplementary Fig. S3a, b). Collectively, circulating naïve immune cells have abundant intracellular ACE2 protein, and cell surface expression of ACE2 can be induced in monocytes and T cells upon activation.

### Cytoplasmic ACE2 rapidly translocates to the cell surface upon TLR activation independent of ACE2 transcription

Next, we sought to investigate the mechanisms by which surface expression of ACE2 was initiated upon immune cell stimulation. We first determined the ACE2 mRNA transcript levels in monocytes stimulated with R848 or LPS. ACE2 mRNA expression remained comparable between sorted ACE2^+^ and ACE2^–^ monocytes stimulated with R848 and was even lower in ACE2^+^ compared to ACE2^–^ monocytes following LPS stimulation (Supplementary Fig. S2a). Similarly, ACE2 mRNA remained comparable between sorted ACE2^+^ and ACE2^–^ T cells following P/I stimulation (Supplementary Fig. S2b).

**Fig. 2:**
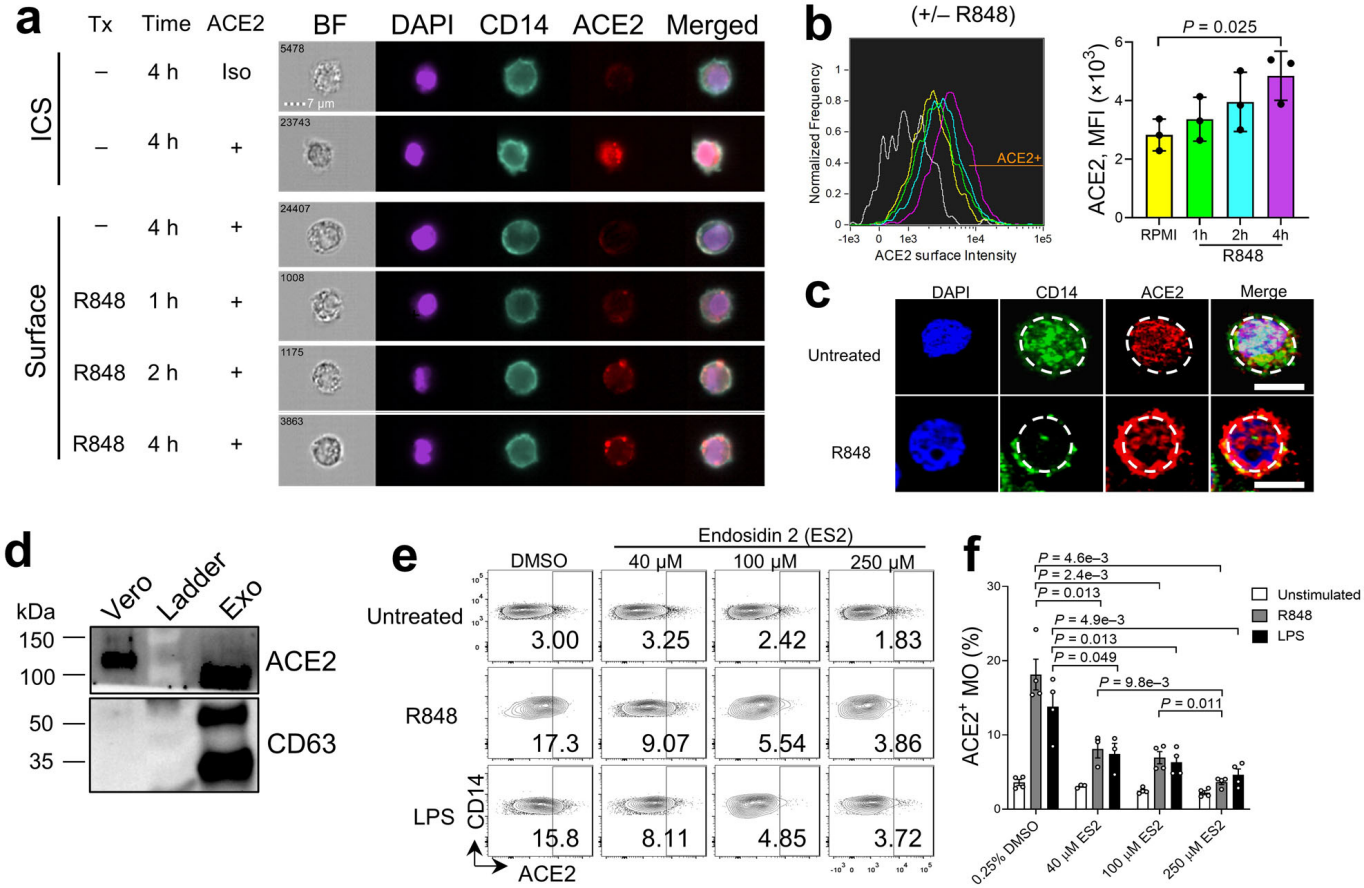
Rapid translocation of cytoplasmic ACE2 to the cell surface of CD14^+^ monocytes after TLR activation. **a** Imaging flow cytometry analysis of cytoplasmic and surface ACE2 protein in CD14^+^ MO with or without R848 treatment up to 4 h. Each cell is represented by the row of images that include bright field (BF), DAPI (purple), CD14 (turquoise), ACE2 (red), and the overlapping image merged with DAPI, CD14, and ACE2. **b** Histograms of ACE2 intensity on the cell surface of CD14^+^ monocytes treated with R848 (left). Grey line represents the isotype control group (Iso). Column graphs indicate MFI of ACE2 surface intensity at other indicated conditions (right) (*n* = 3 biologically independent samples/group). **c** Representative confocal microscopy of ACE2 protein in CD14^+^ monocytes with or without R848 treatment for 24 h. Bars, 10 µm. Dashed lines show location of cell membrane. Images show cells from two healthy donors with similar results. **d** Immunoblot showing ACE2 and CD63 expression in exosomes isolated from plasma of healthy donors. The monkey kidney epithelial cell line Vero was used as a positive control. **e** Flow cytometry analysis of ACE2 surface expression in PBMCs pre-treated with the indicated concentrations of endosidin 2 (ES2) or DMSO for 1–2 h before ex vivo incubation with R484, LPS, or medium alone for 4 h. **f** Frequency of ACE2^+^ MO for data shown in **e**. Bars represent means ± SEM of biologically independent samples (*n* = 3/group for 40 µM ES2 condition; *n* = 4/group for all other conditions).

We next measured ACE2 protein levels by immunoblot in monocytes purified from PBMC with or without R848 treatment and found that the protein levels of ACE2 in monocytes and total PBMCs remained unaltered or slightly decreased (Supplementary Fig. S4), implying that the increase in ACE2 cell surface expression observed in TLR-stimulated monocytes is independent of ACE2 gene transcription and that cytoplasmic ACE2 might translocate to the cell surface upon TLR stimulation. To this end, we applied imaging flow cytometry to monitor protein translocation in blood CD14^+^ monocytes 1 h to 4 h after treatment with R848 or LPS. Consistent with results from conventional flow cytometry (Fig. 1a, b), ACE2 was mainly located in the cytoplasm of resting CD14^+^ monocytes as seen by intracellular staining, while surface ACE2 signals were detected as early as 1 h after R848 or LPS stimulation, with gradual increases over the 2 to 4 h treatment (Fig. 2a, b; Supplementary Figs. S5a, b, and S6). Confocal fluorescence microscopy further confirmed the presence of intracellular ACE2 and CD14 in untreated monocytes and co-localization of ACE2 and CD14 on the monocyte cell surface after R848 treatment (Fig. 2c). These results suggest a rapid translocation of cytoplasmic ACE2 to the monocyte surface upon TLR4/7/8 activation.

### Circulating exosomes contain ACE2 and cellular ACE2 translocation depends on endosomal trafficking

It is possible that ACE2 in mature immune cells may be carried over from early stages of immune cell development and differentiation. To determine the origin of cytoplasmic ACE2 protein in peripheral immune cells, we analyzed ACE2 mRNA expression in 15 clusters identified from CD45^+^ hematopoietic cells in tissues from human embryos, including yolk sac-derived myeloid-biased progenitors, hematopoietic stem and progenitor cells, granulocyte-monocyte progenitors, lymphoid progenitors, monocytes, and macrophages^19^. We found that ACE2 mRNA was detected only in 3 cells of a macrophage subpopulation from a total of 1231 cells (0.24%) among all clusters at the embryonic stage (Supplementary Fig. S7), suggesting that ACE2 protein storage in PBMCs or monocytes is unlikely from the early stage of hematopoietic stem and progenitor cell development.

Extracellular vesicles, including exosomes, have been recognized as a novel mode of intercellular communication and trafficking^20^. Exosomes contain a large cargo of DNA, RNA, and proteins, which can be transferred to both neighboring and distant cells via circulation^21^. We reasoned that ACE2 protein present in monocytes may be derived from ubiquitous ACE2-containing exosomes that have been released by ACE2-expressing cells, such as tissue epithelial cells^22^ and vascular endothelial cells^23^. To test this, we purified circulating exosomes (CD63^+^) from the plasma of healthy donors and found that they indeed contained ACE2 protein (Fig. 2d). Given that the putative destination of exosome-content delivery would be the endosomes^20^, we reasoned that exosome-derived ACE2 may be stored in the endosomes of resting monocytes and could be translocated to the cell membrane through endosomal trafficking upon stimulation. To test this, we pre-treated PBMCs with endosidin 2 (ES2) that binds to the exocyst complex subunit EXO70 and inhibits endosomal recycling. We found that ACE2 surface translocation induced by R848 and LPS treatment was significantly reduced in a dose-dependent manner (Fig. 2e, f). Thus, ACE2 protein stored in CD14^+^ monocytes is likely derived from internalized circulating exosomes harboring ACE2 protein, which is deposited in the monocyte endosome at the steady state and quickly translocates to the cell membrane upon activation.

### TMPRSS2 is localized with ACE2 on the cell surface of monocytes upon TLR stimulation

**Fig. 3:**
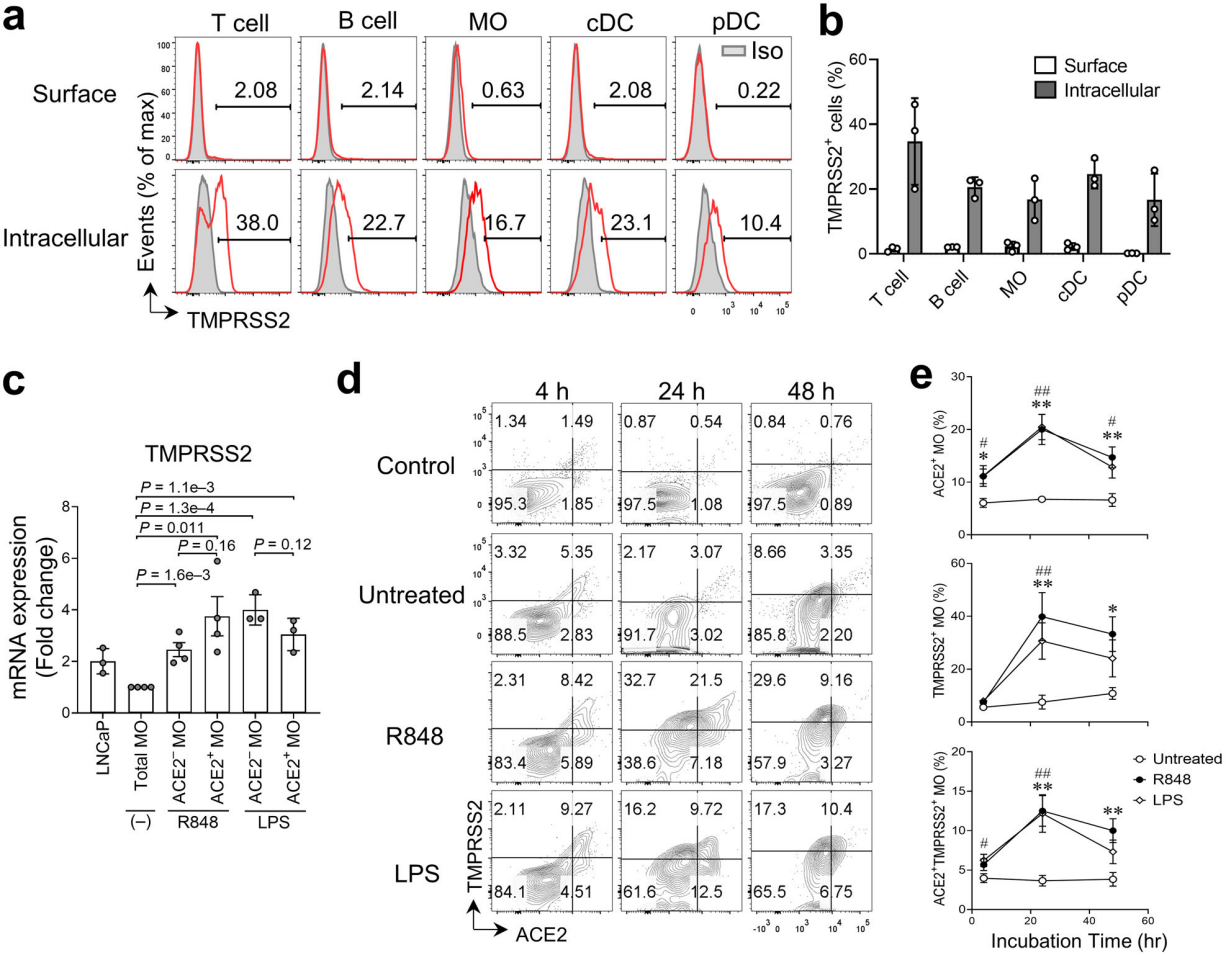
Concurrent expression of ACE2 and TMPRSS2 on the cell surface of monocytes after TLR4/7/8 stimulation. **a** Representative flow cytometric analysis of TMPRSS2 protein expression (red line) in T cells, B cells, CD14^+^ MO, cDCs, and pDCs from PBMCs isolated from healthy donors. Mouse IgG1 antibody (gray tinted) was used as isotype (Iso) control. **b** Frequency of cells with cell surface and cytoplasmic TMPRSS2 expression as in (**a**) (*n* = 3 biologically independent samples/group). **c** Relative TMPRSS2 mRNA expression in indicated cell types compared to expression in total MOs without treatment. (*n* = 3-4 biologically independent samples per group). A human prostate adenocarcinoma cell line LNCaP was used as the positive control. **d** Flow cytometry analysis of ACE2 and TMPRSS2 cell surface expression of CD14^+^ MO in PBMCs cultured ex vivo with R484, LPS, or medium alone (Untreated) for 4, 24, and 48 h. The expression levels of ACE2 and TMPRSS2 in B cells from the same donor served as the untreated baseline control (Control). **e** Frequency of ACE2^+^ MO, TMPRSS2^+^ MO, and ACE2^+^ TMPRSS2^+^ MO for each condition in **d**. Bars represent means ± SEM of biologically independent samples (*n* = 6/group at 4 h and 24 h; *n* = 5/group at 48 h). * indicates comparisons between R848-treated and untreated groups; # indicates comparison of LPS-treated and untreated groups. * or ^#^, *P* < 0.05; ** or ^##^, *P* < 0.01.

SARS-CoV-2 viral entry requires not only binding to the ACE2 receptor, but also S protein priming by TMPRSS2, which cleaves the S protein and permits fusion of the viral and cellular membranes, or endocytosis and cleavage by cathepsin L^2,24^. Unexpectedly, we found that TMPRSS2 was present in about 10%–40% of resting blood cells (Fig. 3a, b). Interestingly, we observed a marked increase in TMPRSS2 mRNA expression in monocytes after R848 or LPS treatment (Fig. 3c), which was comparable in ACE2^+^ and ACE2^‒^ monocytes. Furthermore, TMPRSS2 protein was detected on the cell surface of 5%–10% monocytes following 4 h treatment with R848 or LPS, and the frequency of TMPRSS2^+^ monocytes significantly increased (~20%–50%) at 24 h and 48 h post-treatment compared to untreated groups (Fig. 3d, e). Surface ACE2 and TMPRSS2 were barely detected in B cells even after stimulation with R848 or LPS (Supplementary Fig. S8a). Thus, we used B cells as a baseline control to evaluate the surface expression of ACE2 and TMPRSS2 in monocytes (Fig. 3d). Meanwhile, we observed a robust increase in the frequency of ACE2^+^TMPRSS2^+^ monocytes (~10%–15%) after TLR stimulation for 24–48 h (Fig. 3d, e), suggesting that TLR stimulation induces localization of surface ACE2 and TMPRSS2 protein on monocytes.

Interestingly, although cell surface ACE2 localization was moderately induced (8.9% ± 0.98%, means ± SEM) in cDCs after R848 or LPS treatment, surface expression of TMPRSS2 was almost negligible (3.2% ± 0.87%, means ± SEM) upon stimulation (Supplementary Fig. S8b, c). Very few ACE2^+^TMPRSS2^+^ cDCs (1.1% ± 0.21%, means ± SEM) were detected after R848 or LPS stimulation. Furthermore, contrary to the robust appearance of surface ACE2 in T cells following P/I stimulation, surface TMPRSS2 was hardly detectable (<1%) in activated T cells (Supplementary Fig. S9a, b) and TMPRSS2 mRNA remained comparable between sorted ACE2^+^ and ACE2^–^ T cells upon P/I treatment (Supplementary Fig. S2c). Thus, CD14^+^ monocytes, not cDCs or T cells, co-express surface ACE2 and TMPRSS2 following TLR stimulation, which could be utilized by SARS-CoV-2 for viral entry.

On the other hand, as surface ACE2 and TMPRSS2 were also detected (~5%) in untreated monocytes after 24 h culture (Fig. 3e), we reasoned that they might be induced by certain medium components, such as serum or PBMC-releasing factors. To address this, we analyzed untreated PBMCs and PBMCs treated with R848 or LPS in the presence or absence of serum for 24 h. The frequency of untreated monocytes expressing surface ACE2 and/or TMPRSS2 remained detectable at comparable levels regardless of the presence of serum. However, the R848-treated and LPS-treated monocytes expressed relatively higher levels of surface ACE2 and TMPRSS2 in serum-containing medium than in serum-free medium (Supplementary Fig. S10a, b). These results suggest that surface ACE2 and TMPRSS2 can be enhanced by certain serum nutrient factors when monocytes are activated. The factors driving surface localization of ACE2 and TMPRSS2 in the resting cells appear unrelated to serum nutrients and are possibly certain self-releasing factors produced by the cultured PBMCs.

### ACE2^+^CD14^+^ monocytes are susceptible to infection with SARS-CoV-2 upon TLR activation

**Fig. 4:**
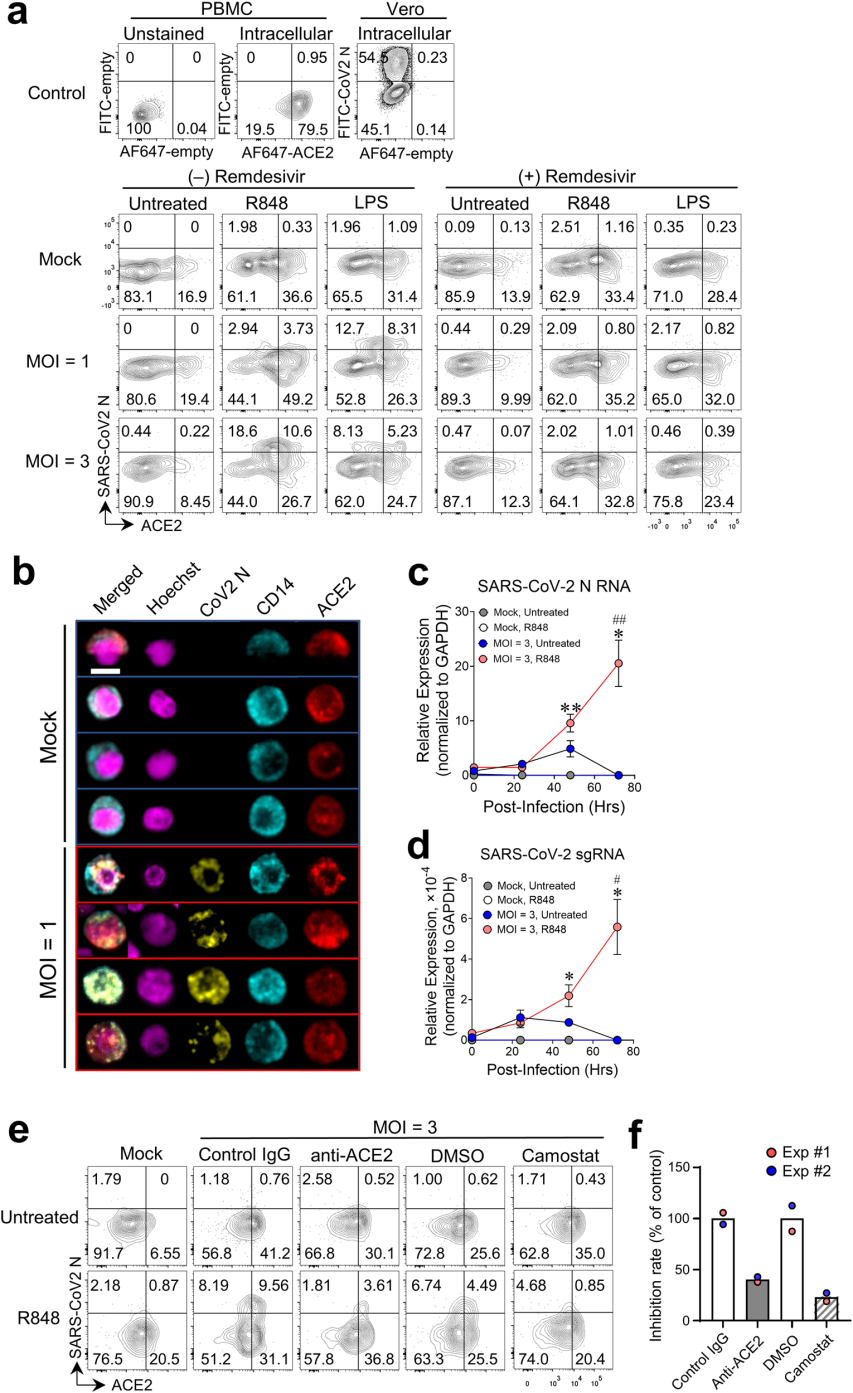
Infection of ACE2^+^CD14^+^ monocytes by SARS-CoV-2 upon TLR4/7/8 activation. **a** PBMCs were cultured with R848, LPS, or medium alone with or without 100 nM remdesivir for 2 h before infection. Pretreated cells were infected with SARS-CoV-2 (MOI = 1 or 3) or mock-infected for 24 h in the presence of stimuli and/or inhibitor. Flow cytometry analysis of CD14^+^ monocytes from mock-infected and SARS-CoV-2-infected cells showing cell surface ACE2 (Alexa Fluor 647-labeled antibody) and intracellular SARS-CoV-2 nucleocapsid (N) protein (FITC-labeled antibody). FACS channels with no staining are shown as “empty”. Data were collected pooled PBMCs from 3 healthy donors. Two independent infection experiments were performed with similar results. **b** Confocal fluorescence microscopy of R848-stimulated PBMCs infected with SARS-CoV-2 as in **a** and stained with Hoechst 33342 (nuclear stain) and fluorescent antibodies against SARS-CoV-2 N protein, CD14, and ACE2. Representative images of cells from mock and infected groups. Scale bar, 5 µm. **c** qRT-PCR of SARS-CoV-2 N RNA in PBMCs pretreated with R848 or medium alone for 2 h and infected with SARS-CoV-2 at MOI = 3 or mock-infected for an additional 2 h. Cells were washed then cultured with fresh medium (10% serum) for indicated time periods before RNA extraction. * indicates comparisons between 0 h and other time points for R848-treated groups with viral infections. # indicates comparison of R848-treated and untreated groups with viral infections at the same time points. * or ^#^, *P* < 0.05; ** or ^##^, *P* < 0.01. **d** qRT-PCR of SARS-CoV-2 sgRNA in the cells as treated in **c**. **e** Flow cytometry of cell surface ACE2 and intracellular SARS-CoV-2 N protein in CD14^+^ monocytes from PBMCs cultured with R848 or medium alone with or without 2 µg/mL anti-ACE2 antibody or goat IgG control, 50 µM camostat mesylate, or 2% DMSO (vehicle) for 2 h and then infected with SARS-CoV-2 at MOI = 3 or mock-infected for 24 h in the presence of stimuli and/or antibody/inhibitor. **f** Percentage of ACE2^+^ CoV-2 N^+^ monocytes in antibody- or inhibitor-treated groups compared to control groups. Each dot represents pooled PBMCs from 3 healthy donors. Two independent infection experiments were performed with similar results.

To determine the physiological significance and functionality of surface expression of ACE2 and TMPRSS2 upon TLR activation in monocytes, we pretreated PBMCs with R848, LPS, or medium alone for 2 h and infected cells with SARS-CoV-2. Infected cells were identified by the presence of cytoplasmic SARS-CoV-2 nucleocapsid (N) protein as measured by flow cytometry. As shown in Fig. 4a, without stimulation, very few (<1%) ACE2^+^CD14^+^ monocytes were infected with the SARS-CoV-2, while the frequency of infected ACE2^+^CD14^+^ monocytes dramatically increased (up to ~10%) after stimulation with R848 or LPS (Fig. 4a). Interestingly, a significant proportion (up to ~18%) of R848 and LPS-treated ACE2^‒^CD14^+^ monocytes were also infected. These results suggest that SARS-CoV-2 could infect blood monocytes through both ACE2-dependent and ACE2-independent mechanisms, which is partially consistent with a recent study^14^. We also observed that few, if any, cDCs were infected by SARS-CoV-2, despite expressing ACE2 on their cell surface upon treatment with TLR4/7/8 ligands (Supplementary Fig. S11).

To assess whether SARS-CoV-2 can actively replicate in the infected monocytes, we treated PBMCs with remdesivir, an inhibitor of the viral RNA-dependent RNA polymerase that suppresses the rapid replication of a range of RNA viruses in human cells, including SARS-CoV-2^25,26^. Treatment with remdesivir had minimal effects on surface ACE2 expression but almost completely blocked SARS-CoV-2 infection of TLR-stimulated ACE2^+^CD14^+^ monocytes and ACE2^‒^CD14^+^monocytes (Fig. 4a). Immunofluorescence staining revealed that punctate viral N protein was mainly located in the cytoplasm of the infected ACE2^+^CD14^+^ monocytes after R848 stimulation (Fig. 4b). Meanwhile, SARS-CoV-2 N RNA and subgenomic RNA (sgRNA) were dramatically increased in R848-treated cells after infection for 48–72 h (Fig. 4c, d). These results suggest that SARS-CoV-2 can replicate in TLR-activated monocytes, which is different from abortive infection of SARS-CoV-2 in monocyte-derived macrophages and monocyte-derived DCs^27^ and is consistent with SARS-CoV-2 active replication in blood neutrophils^28,29^.

Recent evidence has shown that SARS-CoV-2 entry into lung cells can be significantly blocked by treatment with an anti-ACE2 antibody or camostat mesylate, an inhibitor of TMPRSS2^2^. To determine whether co-expression of ACE2 and TMPRSS2 is required for SARS-CoV-2 infection of monocytes, we pretreated PBMCs for 2 h with an anti-ACE2 antibody or an isotype control, or with camostat mesylate or its vehicle control, followed by incubation for 24 h with SARS-CoV-2 at MOI = 3. We observed about 60–80% reduction of the frequency of ACE2^+^CoV-2 N^+^CD14^+^ monocytes after inhibition of ACE2 or TMPRSS2 compared to corresponding controls (Fig. 4e, f). These results suggest that co-expression of surface ACE2 and TMPRSS2 directly contributes to SARS-CoV-2 infection of CD14^+^ monocytes.

### ACE2 surface translocation correlates with hyperactivation and PD-L1 expression in blood myeloid cells

**Fig. 5:**
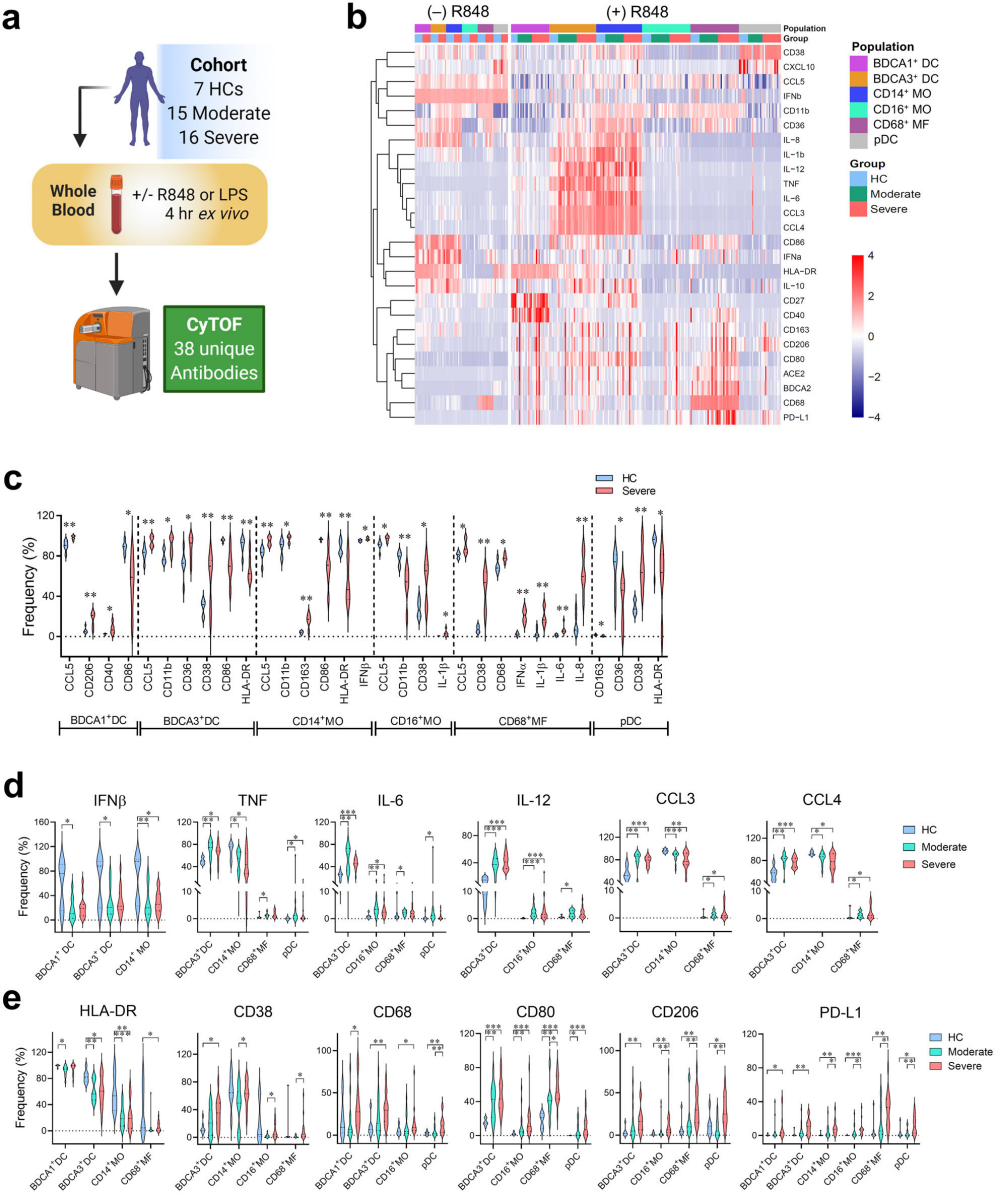
Hyperinflammatory states of blood myeloid cells from COVID-19 patients before and after ex vivo stimulation with R848. Whole blood samples from cohort (*n* = 7 healthy controls (HC); *n* = 15 moderate COVID-19 patients; *n* = 16 severe COVID-19 patients) were stimulated ex vivo with or without R848 for 4 h and then stained with 38 metal-conjugated antibodies for mass cytometry (CyTOF) analysis. **a** Pipeline for processing, treatment, and analysis of blood samples from the cohort. Image generated with BioRender. **b** Heatmap of the frequencies of peripheral blood myeloid populations expressing indicated markers with and without R848 stimulation. **c** Violin plots of the frequencies of myeloid populations expressing indicated markers from HC (*n* = 7) and cells from patients with severe COVID-19 (*n* = 7) without R848 treatment. **d**, **e** Violin plots of frequencies of myeloid populations stimulated ex vivo with R848 expressing indicated cytokines and chemokines (**d**) as well as surface markers (**e**) from HCs (*n* = 7) and COVID-19 patients with moderate (*n* = 15) or severe (*n* = 16) diseases. *P < 0.05; **P < 0.01; ***P < 0.001.

To characterize the myeloid compartment-induced immune responses associated with COVID-19 severity, we stimulated peripheral blood cells from healthy control (HC) and patients with moderate or severe COVID-19 (Supplementary Table S1) for 4 h ex vivo with R848 (Fig. 5a). We identified 6 myeloid populations from peripheral blood based on the expression of lineage markers, including BDCA1^+^ cDCs, BDCA3^+^ cDCs, CD14^+^ classical monocytes, CD14^dim^CD16^+^ nonclassical monocytes, CD68^+^ macrophages, and pDCs (Supplementary Fig. S12a, b) by cytometry by time-of-flight (CyTOF). We found dynamic changes in phenotypic and functional markers in these myeloid populations after R848 treatment (Fig. 5b). Without any treatment, most of the myeloid cell populations from patients with severe COVID-19 disease expressed higher levels of inflammatory markers CCL5, CD11b, CD38 and CD163, and lower levels of HLA-DR and co-stimulatory CD86 than untreated cells from healthy controls (Fig. 5c), which is consistent with the results from another group^30^. Proinflammatory cytokines IL-1β, IL-6, and IL-8 were also highly expressed in untreated CD68^+^ macrophages from patients with severe disease, which is consistent with previously published data^31,32^. After stimulating myeloid blood cells with R848, we observed that multiple myeloid subsets from COVID-19 patients had lower levels of IFNβ and higher levels of TNF, IL-6, IL-12, CCL3, and CCL4 than cells from healthy controls (Fig. 5d). The R848-treated myeloid populations from COVID-19 patients also showed reduced expression of HLA-DR and enhanced expression of CD38, CD68, CD80, and CD206, which was similar to the untreated condition. Interestingly, most myeloid populations from patients with severe COVID-19 expressed higher levels of PD-L1 than cells from moderately ill patients and healthy controls (Fig. 5e).

**Fig. 6:**
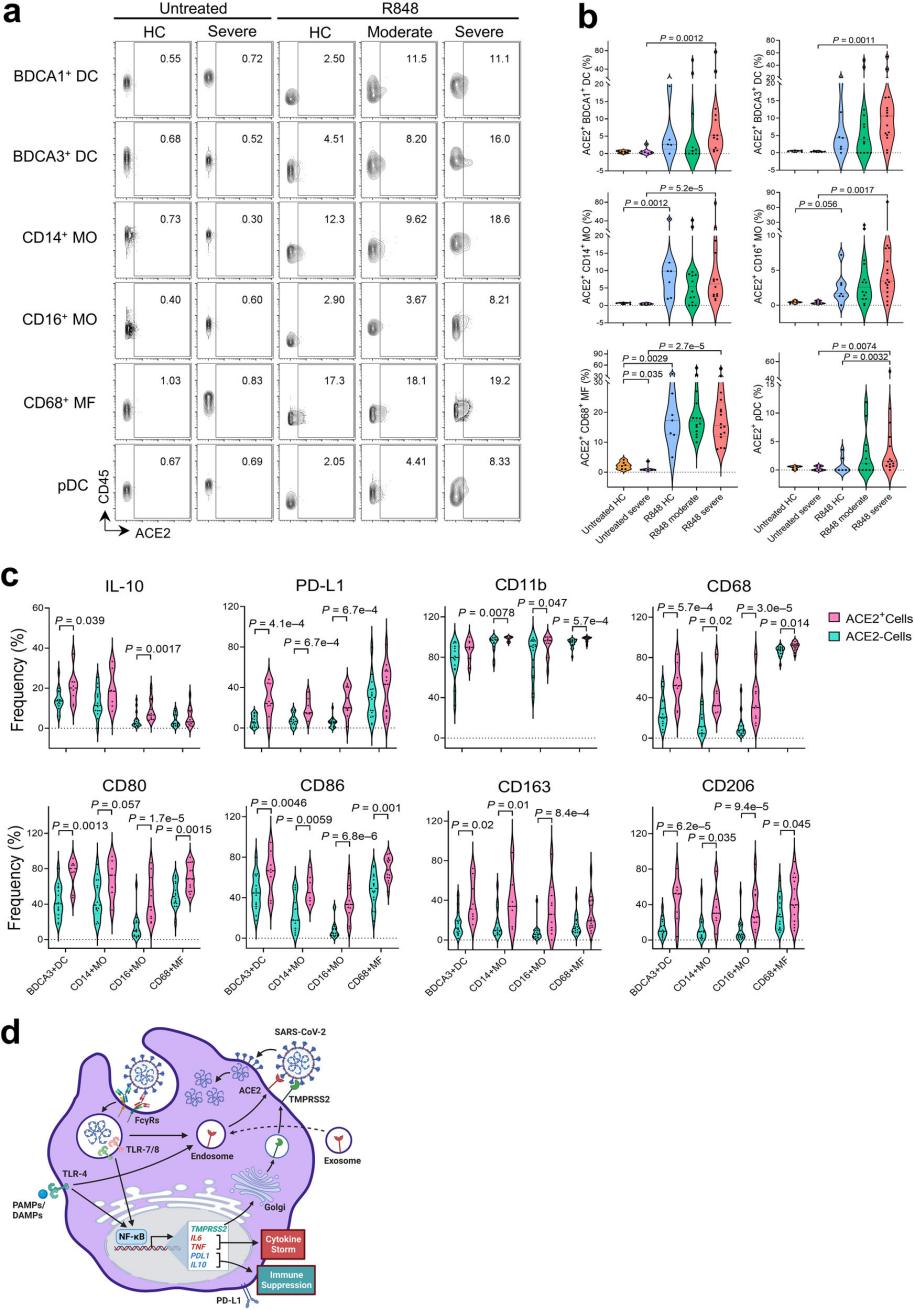
ACE2 surface translocation upon ex vivo R848 stimulation is positively associated with hyperactivation responses and PD-L1 expression in blood myeloid cells from COVID-19 patients. **a** Mass cytometry analysis of cell surface ACE2 expression in blood myeloid populations from untreated samples (*n* = 7 HC; *n* = 7 severe COVID-19) and R848-treated samples (*n* = 7 HC; *n* = 15 moderate COVID-19; and *n* = 16 severe COVID-19). **b** Violin plots of frequencies of the myeloid compartment expressing surface ACE2 as shown in **a**. **c** Frequencies of ACE2^+^ and ACE2^–^ cells (based on ACE2 surface expression) within the myeloid compartment expressing the indicated markers in cells from severe COVID-19 patients. **d** Graphic summary of SARS-CoV-2 infection in monocytes co-expressing surface ACE2 and TMPRSS2 upon TLR4/7/8 activation. ACE2 is taken up by monocytes from ACE2-containing exosomes and stored in the early endosome at steady state. TLR7/8 activation triggered by endocytosis of SARS-CoV-2 or TLR4 activation triggered by viral proteins or host-derived danger signals released during infection stimulates downstream TLR signaling pathways to enhance gene expression of TMPRSS2, proinflammatory cytokines, IL-10, and PD-L1, which drive the cytokine storm and promote immune suppression. TLR4/7/8 activation also induces ACE2 translocation through endosomal trafficking to the cell membrane. Translocated ACE2 and newly synthesized TMPRSS2 at the cell surface facilitate SARS-CoV-2 viral entry and active replication in monocytes. Image generated with BioRender.

We further evaluated surface expression of ACE2 in blood myeloid cell subpopulations from COVID-19 patients and healthy controls before and after R848 ex vivo stimulation. As anticipated, surface expression of ACE2 was barely detected (0.8% ± 0.1%; means ± SEM) in any of the untreated myeloid cells; however, R848 treatment dramatically enhanced surface expression of ACE2 in myeloid subsets from COVID-19 patients and healthy controls, especially in CD14^+^ classic monocytes (11.0% ± 2.7%) and CD68^+^ macrophages (19.3% ± 1.8%) (Fig. 6a, b), which was consistent with our flow cytometry data that showed increased surface ACE2 upon TLR7/8 activation in CD14^+^ monocytes from healthy controls (Fig. 1c, d). Compared to ACE2^–^ cells, ACE2^+^ myeloid cells expressed higher levels of IL-10 and PD-L1, suggesting that ACE2 surface expression is positively associated with immunosuppressive phenotypes (Fig. 6c). Additionally, CD11b, co-stimulatory molecules (CD80 and CD86), and scavenger receptors (CD68, CD163 and CD206) were also upregulated in ACE2-expressing cells, suggesting that ACE2^+^ myeloid cells may have reached a more advanced stage of activation with enhanced phagocytic and migratory capacity^33–35^.

To determine whether TLR4-mediated ACE2 surface translocation in myeloid populations is also associated with COVID-19 severity, we treated peripheral blood cells from the same patient cohorts ex vivo with LPS for 4 h (Fig. 5a). Intriguingly, upregulated surface expression of ACE2 was observed in the major myeloid populations from COVID-19 patients and healthy controls upon ex vivo LPS treatment (Supplementary Fig. S13a), which was coincident with the ACE2 phenotype in R848-treated myeloid cells (Fig. 6b). Importantly, surface expression of ACE2 in BDCA3^+^ cDCs, CD16^+^ monocytes, and pDCs from patients with severe disease was significantly higher than in cells from patients with moderate disease. Additionally, the majority of LPS-treated myeloid populations from patients with severe disease expressed higher levels of PD-L1 than cells from patients with moderate disease and control cells (Supplementary Fig. S13b), coinciding with results from R848 treatment (Fig. 5e). These results collectively indicate that ACE2 cell surface translocation induced by TLR4/7/8 activation positively correlates with hyperactivation and PD-L1 expression in the myeloid cell compartment from COVID-19 patients.

## Discussion

Our current study clearly shows that SARS-CoV-2 can infect blood monocytes that co-express surface ACE2 and TMPRSS2 after stimulation with TLR4/7/8 ligands (Fig. 6d) but cannot infect other immune cells that express only ACE2 upon TLR activation. We uncovered that resting circulating blood cells harbor abundant cytoplasmic ACE2 protein with barely detectable mRNA and cell surface expression, and that circulating ACE2-containing exosomes may be the physiological source of ACE2 cytoplasmic protein in the immune cell compartment. Upon ex vivo activation of myeloid cells with TLR4/7/8 ligands and of T cells with P/I, cytoplasmic ACE2 quickly translocated to the cell surface in most blood immune cells independent of *ACE2* transcription, while elevated TMPRSS2 mRNA and surface expression were present only in monocytes but not in other blood cells. The rapid translocation of ACE2 to the cell surface was blocked by the endosomal trafficking inhibitor endosidin 2. Monocytes with cell surface ACE2 and TMPRSS2 were efficiently infected by SARS-CoV-2, and infection was almost completely blocked by the viral replication inhibitor remdesivir, anti-ACE2 antibodies, and the TMPRSS2 inhibitor camostat.

Our data and two recent studies from Sefik^15^ and Junquira^14^ all indicate that blood monocytes and macrophages can be infected by SARS-CoV-2. Sefik showed that ACE2 surface expression was higher in infected human macrophages and that ACE2 blockade significantly diminished infection in these cells, suggesting that ACE2 can mediate viral entry into human lung macrophages, supporting our results. But that study did not explain the origin of cell-surface ACE2 protein, since ACE2 mRNA is rarely detected in human lung macrophages. Our study has revealed that exosome-derived cytoplasmic ACE2 can translocate to the cell surface of most blood immune cells upon stimulation. Junquira’s group observed that LPS did not induce ACE2 expression in monocytes and that blocking ACE2 and TMPRSS2 did not diminish monocyte infection, which was FcγR dependent. However, our study demonstrated that TLR ligands including LPS could induce cell surface ACE2 in monocytes and ACE2 and TMPRSS2 blockage partially inhibits monocyte infection by SARS-CoV-2. The discrepancy between Junqueira’s study^14^ and our results could be due to different serotypes (*E.coli* O55:B5 (Sigma Aldrich, L6529) vs *E.coli* O111:B4) and doses (100 ng/mL vs 1 µg/mL) of LPS applied in the two studies, respectively. To rule out this possibility, we treated PBMCs from two healthy donors with above LPS serotypes and concentrations overnight (16 h) and found dramatic upregulation of ACE2 surface expression in monocytes following LPS treatments with both LPS serotypes and concentrations compared to isotype and non-treated groups (Supplementary Fig. S14). These results suggest that LPS serotypes O55:B5 and O111:B4 at as low as 100 ng/mL could sufficiently trigger surface translocation of ACE2 in the treated monocytes, at least in this study. Another possibility for this discrepancy could be the different culture conditions for monocyte stimulation. In Junqueira’s study, monocytes were purified from PBMCs and then stimulated with LPS. Instead, we directly stimulated PBMCs with LPS and then analyzed monocytes’ ACE2 expression. It is possible that the existence of support factors or cells from PBMCs is indispensable for surface translocation of ACE2 in monocytes upon TLR stimulation. It would be interesting to further determine whether cell–cell contacts or soluble factors released by the activated supporter cells are required for this process. We also noticed a discrepancy in the infection rate of CD14^+^ monocytes by SARS-CoV-2 after LPS stimulation between Junqueira’s study^14^ and our results. In their study, blood CD14^+^CD16^‒^ classical monocytes from COVID-19 patients did not express surface ACE2 upon LPS stimulation and failed to be infected by SARS-CoV-2. However, we found that CD14^+^ monocytes were efficiently infected by SARS-CoV-2 after ex vivo stimulation with TLR ligands, which was mediated by rapid surface translocation of ACE2 and TMPRSS2. The difference between our data and Junquira’s data could be due to different flow cytometry gating strategies (Supplementary Fig. S12). Our CD14^+^ monocyte population actually contained CD14^+^CD16^+^ intermediate monocytes, which were shown to be infected by SARS-CoV-2 in Junquira’s study. When we further analyzed ACE2 and CD16 surface expression in the CD14^+^ monocytes by our gating strategy, we indeed found co-expression of ACE2 and CD16 in CD14^+^ monocytes at a variety of levels from healthy control and COVID-19 patients (Supplementary Fig. S15a, b). These results suggest that the successful infection of SARS-CoV-2 in CD14^+^ monocytes may be partially due to the existence of CD14^+^CD16^+^ intermediate monocytes in this population.

Additionally, TLR4/7/8-activated peripheral myeloid cells from patients with moderate to severe COVID-19 produced less IFNβ and more proinflammatory cytokines and had higher PD-L1 expression than healthy control cells. Importantly, ACE2 surface translocation was positively associated with hyperactivation and IL-10 and PD-L1 expression in myeloid cells from COVID-19 patients. Although the role of checkpoint molecules in the context of acute and chronic infection is less defined, the PD-1/PD-L1 pathway appears to permit pathogens to escape elimination by suppressing immune responses through inhibition of T cell effector function, possibly resulting in chronic infection^36^. Recent studies have shown increased expression of checkpoint molecule PD-1 in CD8 T cells, invariant natural killer T cells, and mucosal-associated invariant T cells from patients with moderate to severe COVID-19^37–39^. Thus, increased IL-10 and PD-L1 expression in myeloid cells may downregulate antiviral immune activity in CD8 T cells and unconventional T cells.

There are some limitations in our current study, including that all experiments were performed ex vivo. It remains unknown why untreated PBMCs isolated from COVID-19 patients did not show relatively high levels of surface ACE2 protein. One possibility is that ACE2 translocation is an early event during infection, and infected cells quickly migrate to local organs or quickly undergo pyroptosis^14^. A recent preprint study showing that long SARS-CoV-2 nucleocapsid (N) RNA transcripts were identified in PBMCs from 2 of 11 COVID-19 patients as early as 2 to 6 days after hospital admission^40^. Junqueira’s study^14^ also showed SARS-CoV-2 N proteins were identified in two minor monocyte subsets (intermediate and non-classical) isolated from 12 COVID-19 patients, and ACE2 surface protein levels were significantly increased in monocytes purified from 4 COVID-19 patients. Although we did not catch the early infection period of our COVID-19 patients, the evidence provided by at least these two studies supports our working hypothesis of ACE2-mediated SARS-CoV-2 infection in the monocytes. Furthermore, it remains unclear why TMPRSS2 surface expression upon TLR stimulation seems to occur specifically in monocytes and not in other blood cells. Deeply exploring the transcriptomes and accessible chromatin landscapes during TLR activation in each type of blood cell using single-cell technologies (e.g., scRNA-seq, CITE-seq, scATAC-seq) could address the above question and might identify specific subsets of monocytes that express both ACE2 and TMPRSS2. It would also be interesting to see if inhibition of ACE2 surface translocation could reduce the hyperinflammation and T cell exhaustion typically observed in severely ill patients with COVID-19.

Collectively, our results show that TLR4/7/8-induced translocation of ACE2 from endosomes to the cell surface alongside TMPRSS2 cell surface expression promotes a viable route for SARS-CoV-2 infection in circulating monocytes. Currently, most ACE2-based therapeutic strategies aim to tackle the virus using ACE2 inhibitors^41^ or exogenous soluble ACE2 for virus neutralization^42^, which do not directly reduce membrane availability. Thus, an understanding of ACE2 subcellular localization and trafficking is essential for investigating the potential of ACE2 as an effective therapeutic target for COVID-19. Our work not only provides a new mechanism of SARS-CoV-2 pathogenesis, but also unveils a prospective therapeutic strategy of targeting ACE2 membrane trafficking for preventing monocyte/macrophage infection.

## Materials and methods

### Human subjects

Blood samples were obtained from individuals who provided written informed consent and samples were deidentified prior to processing under guidelines approved by the Henry Ford Health System Institutional Review Board. Donors with SARS-CoV-2 viral infection were enrolled in Detroit, MI, including subjects with moderate or severe disease following Centers for Disease Control and Prevention criteria as reported previously^43^. In general, patients with moderate disease presented with cough, fever, myalgia, dyspnea, SaO_2_ < 94% on room air at rest or with exertion (walking); were shown to have pulmonary infiltrates by radiology imaging; and needed supplemental O_2_ therapy. Patients with severe disease had all above symptoms and had respiratory failure requiring mechanical ventilation. Identification of SARS-CoV-2 RNA from patient specimens was performed using PCR methods that were validated against the CDC reference method in Henry Ford’s Microbiology Core Laboratory. The plasma from healthy donors (control) was measured for IgG antibodies to SARS-CoV-2 receptor binding domain Spike protein (RBD-S) using a Dxl 800 automated immunoassay analyzer (Beckman Coulter, Brea, CA). One sample from the healthy donors showed a positive result, indicating that one asymptomatic, previously infected individual was included in the healthy control group. All blood samples were collected in cell preparation tubes (CPT tubes, BD Biosciences, San Jose, CA) containing sodium heparin and were processed within 4 h of collection. All assays for mass cytometry used fresh blood cells, and the assays for flow cytometry used either fresh or frozen PBMCs.

### Cell isolation and stimulation

For ex vivo stimulation assays analyzed by CyTOF, 250 µL of heparinized whole blood cells from CPT tubes were transferred to a 5 mL round-bottom tube with a lid (Thermo Fisher Scientific, Waltham, MA), followed by addition of RPMI medium (Sigma Aldrich, St. Louis, MO), resiquimod (R848, InvivoGen, San Diego, CA), or lipopolysaccharide (LPS, Sigma Aldrich, L4391) at a final concentration of 1 µg/mL. Brefeldin A (eBiosciences^TM^, Thermo Fisher Scientific) and monensin (eBiosciences^TM^, Thermo Fisher Scientific) were simultaneously added to the cells at a final concentration of 3 µg/mL and 2 µM, respectively. The cells were then incubated for 4 h at 37 °C with 5% CO_2_. After stimulation, 50 µL of metal-conjugated surface antibody mixture was added to the tubes followed by incubation at 4 °C for 30 min. Next, 420 µL of PROT1 Proteomic Stabilizer (Smart Tube Inc., Las Vegas, NV) was added to the cells with incubation at room temperature (RT) for 10 min. The stained fixed cells were immediately placed at −80 °C for storage.

For ex vivo stimulation assays analyzed by flow cytometry, fresh PBMCs were isolated from CPT tubes using a standard protocol^44^. Briefly, whole blood cells were diluted with Ca^2+^-free and Mg^2+^-free PBS buffer (Corning, NY) containing 2% fetal bovine serum (FBS, R&D Systems, Minneapolis, MN) and 2 mM EDTA (VWR Life Science, Radnor, PA) and slowly overlaid onto Ficoll-Paque™ (GE healthcare, Chicago, IL) at 2:1 ratio by volume. The samples were centrifuged at RT for 20 min at 800× *g* with no braking. Next, the mononuclear cell layer at the plasma-Ficoll interface was moved into a new 50 mL tube and washed with PBS buffer and red blood cell lysis buffer at RT for 5 min. The isolated PBMCs were used on the day of isolation or placed at −80 °C for storage. Two million fresh or thawed PBMCs were transferred to each well of a round-bottom 96-well culture plate (Thermo Fisher Scientific) in 200 µL of RPMI medium containing 10% FBS. The thawed PBMCs were recovered for 2 h at 37 °C with 5% CO_2_. In some experiments, PBMCs were treated with different concentrations of endosidin 2 (ES2, Sigma Aldrich) or 0.25% DMSO (the vehicle for ES2 at the highest concentration; ATCC, Manassas, VA) for 1–2 h during recovery stage. The fresh and recovered cells were then cultured with 1 µg/mL R848, LPS, or medium alone for 4 h. For TCR-independent T cell stimulation, PBMCs were cultured in RPMI medium containing 10% FBS in the presence of phorbol 12-myristate 13-acetate (PMA) (50 ng/mL) and ionomycin (1 μM) for a total of 4 h at 37 °C. After incubation, the cells were washed with wash buffer in PBS containing 2% FBS and 1 mM EDTA prior to staining.

### Mass cytometry (CyTOF)

The metal-conjugated antibodies used for CyTOF were purchased (Fluidigm or The Longwood Medical Area CyTOF core, Boston, MA) or conjugated in house using MaxPar X8 labeling kits according to the manufacturer’s instructions (Fluidigm) (Supplementary Table S2). The frozen stained blood samples were placed at RT for 30–50 min until fully thawed then incubated with 1× Thaw-Lyse buffer (Smart Tube Inc.) at RT for 10 min. The lysis steps were repeated a few times until the pellet turned white. If intracellular staining was needed, the cells were washed with Maxpar Cell Staining Buffer (Fluidigm), permeabilized with BD Perm II working solution (BD Biosciences) at RT for 10 min, and incubated with intracellular antibody cocktail containing 100 U/mL heparin solution (Sigma–Aldrich) at 4 °C for 45 min. The cells were then incubated with Maxpar Fix and Perm Buffer (Fluidigm) containing 125 nM Cell-ID Intercalator-Ir solution (Fluidigm) at 4 °C overnight or up to 48 h before sample acquisition. Groups of samples (10–16/day) were assessed by Helios mass cytometry (Fluidigm) in 16 independent experiments using a flow rate of 45 µL/min in the presence of EQ Calibration beads (Fluidigm) for normalization. An average of 360,000 ± 13,600 cells (means ± SEM) from each sample were acquired and analyzed by Helios. Gating was performed on the Cytobank platform (Cytobank, Inc. Santa Clara, CA) and FlowJo 10.5.3 (BD Biosciences).

### CyTOF data processing

All FCS files generated by CyTOF were normalized and concatenated, if necessary, using CyTOF Software version 6.7. All CyTOF processed files were also uploaded to the cloud-based Cytobank platform and beads, debris, doublets, and dead cells were manually removed by sequential gating shown in Supplementary Fig. S12. The CD45^+^CD66b^–^ live singlets were either selected for viSNE^45^ analysis or gated manually with multiple cell lineage markers to define immune populations. The expression of checkpoint molecules and functional markers of each identified immune population was further analyzed by Cytobank platform or FlowJo software. The immune populations gated for each sample with less than 15 events were eliminated from the functional analysis. Heat maps and other plots were generated using Cytobank platform, GraphPad Prism 8.4.3 software (GraphPad, La Jolla, CA), or R 4.0.2 packages.

### Flow cytometry

Single-cell suspensions were centrifuged at 450× *g* for 7 min, resuspended in ice-cold staining buffer (1× PBS with 2% FBS), and placed in a 96-well round-bottom plate (Thermo Fisher Scientific). After incubation with human Fc receptor blocking solution (BioLegend, San Diego, CA) at 4 °C for 15 min, cells were stained with a mixture of fluorescent surface antibodies at 4 °C for 30 min. For intracellular staining, the cells were fixed with IC Fixation Buffer (eBiosciences^TM^, Thermo Fisher Scientific) at 4 °C for 30 min followed by incubation with fluorescent intracellular antibody cocktail in 1× Intracellular Fixation & Permeabilization Buffer (eBiosciences^TM^, Thermo Fisher Scientific) at 4 °C for 30 min. The full list of fluorescent antibodies used is in Supplementary Table S3. DAPI was added to the unfixed cells with surface staining at a final concentration of 1 μg/mL immediately before sample acquisition. The stained samples were then acquired on a FACSCelesta^TM^ flow cytometer (BD Biosciences) using BD FACSDiva software version 8.0.2 (BD Biosciences). All data were analyzed using FlowJo 10.5.3 (BD Biosciences). In some experiments, cells were sorted by a FACSAria^TM^ II flow cytometer (BD Biosciences) with >95% purity.

### Imaging flow cytometry

PBMCs were stained with anti-CD3/CD19/CD56-FITC, anti-CD14-PE, and anti-ACE2-Alexa Fluor 647 antibodies. DAPI (1 μg/mL) was used for nuclear imaging. Normal mouse IgG1-Alexa Fluor 647 was used as isotype control. In total, 200,000–400,000 events were collected for all samples on an ImageStream IS100 using 405 nm, 488 nm, and 642 nm laser excitation. Cell populations were hierarchically gated for single cells that were in focus, as described previously^46^, and were positive for CD14 and negative for CD3, CD19, and CD56, which were defined as CD14^+^ monocytes. After the gates were applied, a total of 3000–5000 CD14^+^ monocytes were acquired for each sample and incorporated into the final analysis. Following data acquisition, the surface expression of ACE2 was measured by calculating the intensity feature of ACE2 signals using membrane mask in the IDEAS software package^46^.

### Virus infection

All SARS-CoV-2 infection-related work was performed in a Biosafety level 3 (BSL3) facility at the University of Michigan under the guidance of the Centers for Disease Control and Prevention. LNCaP, Vero E6, and Huh-7 cell lines were maintained in DMEM supplemented with 10% FBS, 100 U/mL penicillin, 100 U/mL streptomycin, and glutamine at 37 °C with 5% CO_2_. All cell lines tested negative for mycoplasma. SARS-CoV-2 strain WA1/2020 (BEI resources, Catalog #NR-52281) was added in the BSL3 containment facility at a final working dilution equivalent to a multiplicity of infection (MOI) of 1 and allowed to incubate for 24 h at 37 °C. Frozen PBMCs were thawed and seeded at 1 × 10^6^ cells/well on a 12-well cell culture plate. After resting at 37 °C with 5% CO_2_ for 2 h, cells were cultured in the presence or absence of 1 µg/mL R848 (InvivoGen, Catalog #tlrl-r848, Dan Diego, CA), 1 µg/mL LPS (Sigma-Aldrich, Catalog #L4391), or 100 nM Remdesivir (MedChemExpress, Catalog #GS-5734-D5) for another 2 h at 37 °C. After the second 2 h incubation, SARS-CoV-2 was then added to the cells at an MOI of 1 or 3 and allowed to incubate for 24 h at 37 °C in the presence of stimuli and/or inhibitor.

The infected Vero E6 and Huh-7 cells were treated with trypsin, blocked with human Fc Block, fixed with 4% paraformaldehyde (PFA), and resuspended in PBS. The infected PBMCs were blocked with PBS containing 3% BSA and human Fc receptor blocking solution and stained with fluorescent antibodies against surface ACE2, CD3, CD11c, CD123, CD14, and CD19 as listed in Supplementary Table S3 and fixed with 4% PFA. Tubes containing fixed infected cells were sealed and decontaminated, and samples were transferred to a BSL2 lab. The purified SARS-CoV-2 nucleocapsid (N) antibody (Antibodies-online Inc., ABIN6952432, Limerick, PA) was labeled with the FITC conjugation kit—lightning-link (Abcam, ab102884). The fixed cells were then permeabilized and stained with the conjugated SARS-CoV-2 N-FITC antibody (1:400) in 1× Intracellular Fixation & Permeabilization Buffer (eBiosciences^TM^) at 4 °C for 30 min prior to flow cytometry analysis.

### Exosome isolation and purification

Plasma exosomes were isolated and purified with ExoQuick ULTRA EV Isolation Kit (SBI System Biosciences, Palo Alto, CA), according to the manufacturer’s protocol. Briefly, 67 µL of ExoQuick was added to 250 µL plasma after debris was removed and then incubated on ice for 30 min and centrifuged at 3000× *g* for 10 min. The pellet was resuspended and loaded to a column for purification.

### Immunofluorescence microscopy

PBMCs were blocked with human Fc Block, stained with ACE2-Alexa Fluor 647 (1:100) and CD14-PE (1:30) antibody, fixed with 4% PFA, permeabilized with Perm buffer, and stained with SARS-CoV-2 N-FITC antibody containing Fc Block. Tubes were sealed, the surface was decontaminated, and samples were transferred to a BSL2 lab. Cells were incubated with Hoechst 33342 (Invitrogen, 1:2000) and centrifuged (180× *g*) into a 384-well plate (Perkin Elmer) in PBS for imaging. A Yokogawa Cell Voyager 8000 high content microscope was used for automated 4-color imaging. A 40X/1.0 NA water immersion objective was used with a 50 µm spinning disk confocal unit. The 405 nm/488 nm/561 nm/640 nm laser lines and corresponding emission filters (445/45 nm, 525/50 nm, 600/37 nm, 676/29 nm) were used to capture Hoechst-33342, FITC, PE, and Alexa Fluor-647, respectively. Maximum projection images were collected over a 10 µm range with a 0.3 µm step size. A total of 80 fields per well were collected and CellProfiler^47^ was used to identify cells and then classify as CD14 positive by intensity measurements. Color images were produced in FIJI^48,49^ and brightness/contrast was optimized per channel and was held constant for all cell images shown.

For confocal imaging analysis of ACE2 in CD14^+^ monocytes, PBMCs were treated with 1 µg/mL R848 or medium alone for 24 h and stained with biotin-conjugated CD14 antibody followed by CD14^+^ monocyte enrichment using Streptavidin Magnetic Beads (Thermo Scientific). The enriched monocytes reached over 85% purity and were immobilized onto slides using Cytospin^TM^ 4 Cytocentrifuge (Thermo Scientific). The mounted cells were immediately fixed by IC Fixation Buffer at RT for 30 min, washed by PBS once, and blocked by human Fc block at RT for 30 min. The slides were then incubated with ACE2-Alexa Fluor 647 (1:50) antibody in PBS containing 3% BSA at 4 °C overnight. After 4 times of washes with PBS, the slides were incubated with Alexa Fluor 488-conjugated streptavidin (1:1000) at RT for 1 h and then stained with DAPI (0.5 µg/mL) at RT for 2 min. Images were captured on an Olympus FV1000 confocal microscope using the 405 nm/473 nm/635 nm laser lines and corresponding emission wavelengths (461 nm, 520 nm, and 668 nm).

### RNA extraction and quantitative real-time PCR

Total RNA was extracted with GenElute, purified with the Total RNA Purification Kit (Sigma Aldrich), and reverse-transcribed to cDNA with High Capacity cDNA Reverse Transcription Kits (Applied Biosystems, Foster City, CA). Quantitative real-time PCR (qRT-PCR) reactions were prepared using FastStart Universal SYBR Green Master (ROX, Roche) and carried out using QuantStudio 7 Flex Real-Time PCR System (Applied Biosystems). Data were collected using QuantStudio 7 Flex Real-Time PCR System software version 1.2 (Applied Biosystems) and analyzed using Microsoft Excel 2016 (Microsoft, Redmond, Washington). Quantitative gene expression data were normalized to GAPDH expression. The following primers were used: human ACE2: forward 5′-TGAAGTTGAAAAGGCCATCAG-3′ and reverse 5′-GAGGTCCAAGTGTTGGCTGT-3′; human TMPRSS2: forward 5′-GAGAAAGGGAAGACCTCAGAAG-3′ and reverse 5′-GGTGTGATCAGGTTGTCATAGA-3′; human GAPDH: forward 5′-CCTGCACCACCAACTGCTTA-3′ and reverse 5′-GGCCATCCACAGTCTTCTGAG-3′; SARS-CoV-2 N1 nucleocapsid gene: forward 5′-GACCCCAAAATCAGCGAAAT-3′ and reverse 5′-TCTGGTTACTGCCAGTTGAATCTG-3′. SARS-CoV-2 sgRNA: forward 5′-GTAACAAACCAACCAACTTTCG-3′ and reverse 5′-CATTGTTCACTGTACACTCGATC-3′.

### Immunoblot

Total protein was isolated using radioimmunoprecipitation assay (RIPA) buffer (Thermo Fisher Scientific, Waltham, MA). Equal amounts of protein were separated on 12% sodium dodecyl sulfate polyacrylamide gels and electro-transferred onto nitrocellulose membrane (Bio-Rad) at 100 V for 2.5 h at 4 °C. The membrane was blocked with 5% BSA for 1 h at RT and probed with primary antibodies against ACE2 (1:600, AF933, R & D Systems) and TMPRSS2 (1:1000, sc-515727, Santa Cruz). GAPDH (1:1000, 3683 S, Cell Signaling) was used as internal control. HRP-conjugated rabbit anti-goat (1:2000, 1721034, Bio-Rad) or goat anti-mouse (1: 2000, STAR117, Bio-Rad) were used as secondary antibodies. For immunoblotting of exosomal proteins, rabbit polyclonal CD63 antibody (1:1000, Abcam) and goat-anti-rabbit (1:2000, Cell Signaling) were used. Target proteins were visualized with an enhanced chemiluminescence detection system (GE Healthcare, NJ) using ChemiDocTM MP imaging system and associated software (Bio-Rad, Hercules, CA).

### Statistics and reproducibility

No statistical method was used to predetermine sample sizes. All data were collected from at least two independent experiments. For categorical data, the two-tailed Fisher’s exact test was used. For all continuous independent variables, if the data were not normally distributed as tested by the Shapiro–Wilk test, a nonparametric Mann–Whitney *U* test was used. For normally distributed data, if variances were equal, the Student’s unpaired two-tailed *t*-test was used; otherwise, the unpaired two-tailed *t* test with Welch’s correction was used. For pairwise comparison of two groups, the two-tailed paired *t*-test was used. Statistical analyses were performed with GraphPad Prism 8.4.3 software or R 4.0.2 packages. Statistical significance is displayed as: *P < 0.05; **P < 0.01; ***P < 0.001.

## Supplementary information

Supplementary Information

## Data Availability

The datasets generated in this article (and its Supplementary Information files) are available from the corresponding authors upon reasonable request.
