# Supplementary material for: Surface translocation of ACE2 and TMPRSS2 upon TLR4/7/8 activation is required for SARS-CoV-2 infection in circulating monocytes
Source: Cell Discov. 2022 Sep 9;8:89. doi: 10.1038/s41421-022-00453-8 (PMC9462622; doi:10.1038/s41421-022-00453-8)
Supplement: Supplementary file 1 — Supplementary Information [file 41421_2022_453_MOESM1_ESM.pdf]

Supplementary Information

Surface translocation of ACE2 and TMPRSS2 upon TLR4/7/8 activation is required for SARS-CoV-2 infection in circulating monocytes

Yao, Subedi, et al.

Supplementary Figure S1

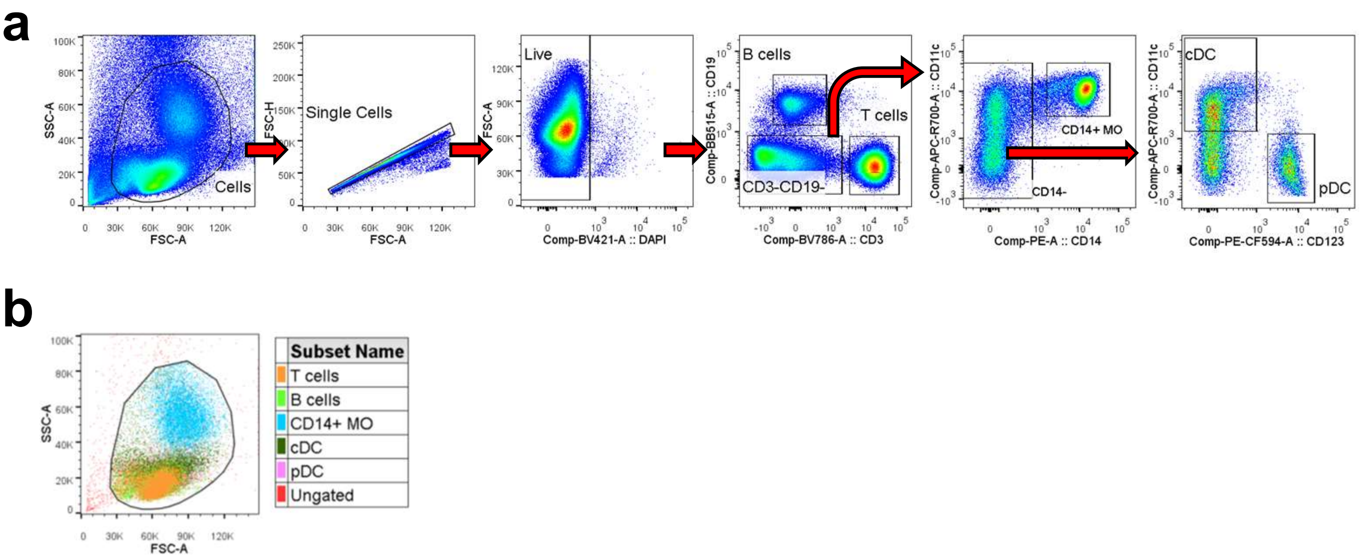

Supplementary Figure S1

Flow cytometry gating strategy of PBMC major populations.

(**a**) Gate strategy of B cells, T cells, CD14^{+} monocytes (MO), cDCs and pDCs in PBMCs freshly isolated from healthy subjects. (**b**) Overlaid scatterplot of each PBMC subset from **a**.

Supplementary Figure S2

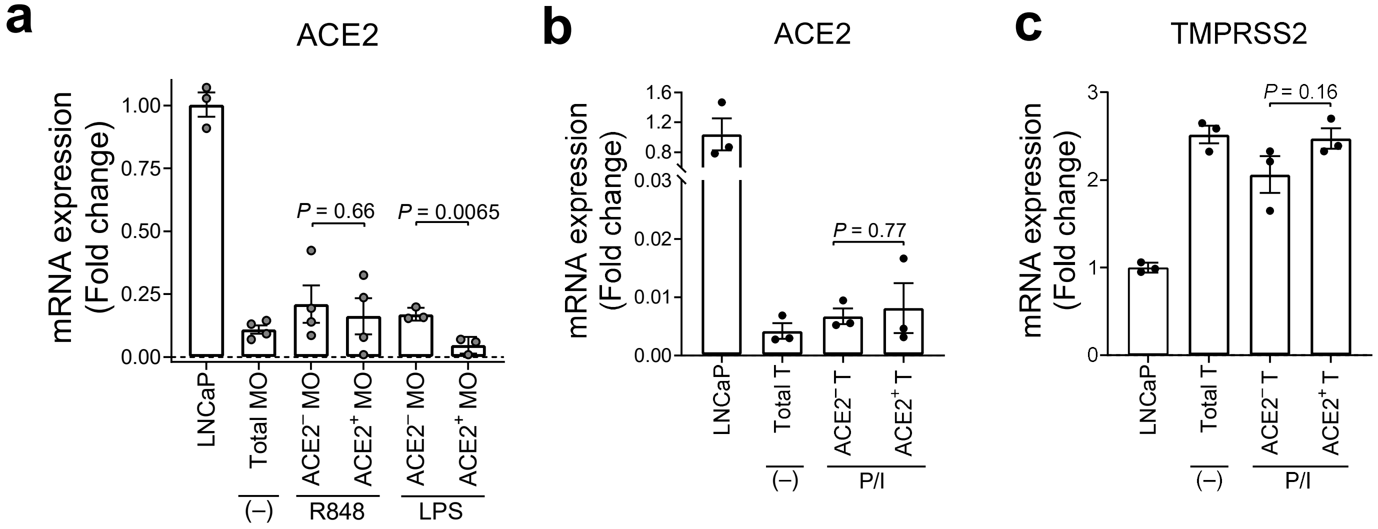

Supplementary Figure S2

mRNA expression of ACE2 and TMPRSS2 in monocytes and T cells upon ex vivo stimulation.

(**a**) PBMC samples were ex vivo cultured in the presence or absence of R848 or LPS for 4 h and stained with anti-CD14 and anti-ACE2 antibodies on cell surface. $ACE2^{+}CD14^{+}$, $ACE2^{-}CD14^{+}$ and total monocytes (MO) were sorted for qPCR analysis. Relative expression of ACE2 mRNA in the indicted monocyte populations compared to that in human prostate adenocarcinoma LNCaP cell line. N = 3-4 biologically independent samples per group. (**b, c**) PBMCs were ex vivo cultured with PMA plus ionomycin for 4 h. $ACE2^{+}$ $CD3^{+}$ T, $ACE2^{-}CD3^{+}$ T and total $CD3^{+}$ T cells were sorted for qPCR analysis. (**b**) Relative expression of ACE2 mRNA in the indicted T cell populations compared to that in LNCaP cells. N = 3 biologically independent samples per group. (**c**) Relative expression of TMPRSS2 mRNA in the indicated populations compared to that in the total T cells without treatment. N = 3 biologically independent samples per group.

Supplementary Figure S3

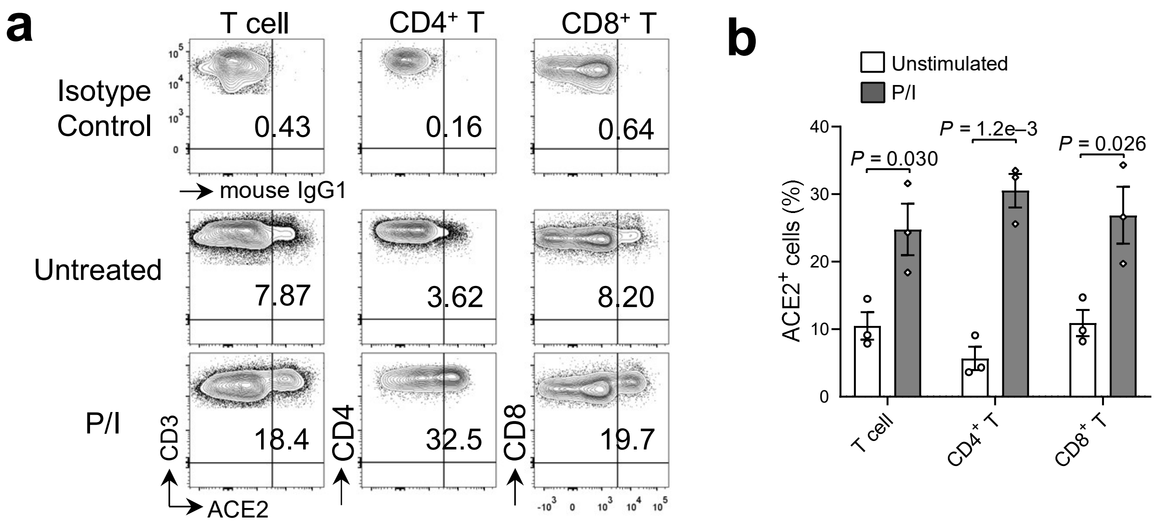

Supplementary Figure S3

ACE2 surface expression in T cell populations from PBMCs.

(**a**) PBMCs were cultured ex vivo in the presence of PMA plus ionomycin (P/I) for 4 h, followed by flow cytometry analysis of surface ACE2 in total T cells and CD4$^+$ and CD8$^+$ T cells. (**b**) The frequencies of the indicated T cell populations expressing surface ACE2 as in **a** (n = 3 biologically independent samples/group).

Supplementary Figure S4

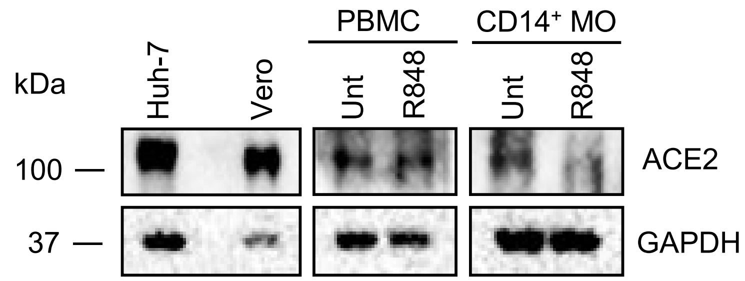

Supplementary Figure S4

ACE2 protein levels in PBMCs and monocytes upon ex vivo stimulation with R848.

PBMCs isolated from healthy donors were ex vivo cultured in the presence or absence of R848 for 24 h followed by $CD14^+$ monocyte (MO) enrichment (>85% purity). A total of 60 µg protein from untreated (Unt) or R848-treated PBMCs and enriched monocytes were loaded to the gels for immunoblotting. Representative blot bands showing the expression of ACE2. GAPDH serves as a loading control. Huh-7 and Vero cell lines served as positive controls.

Supplementary Figure S5

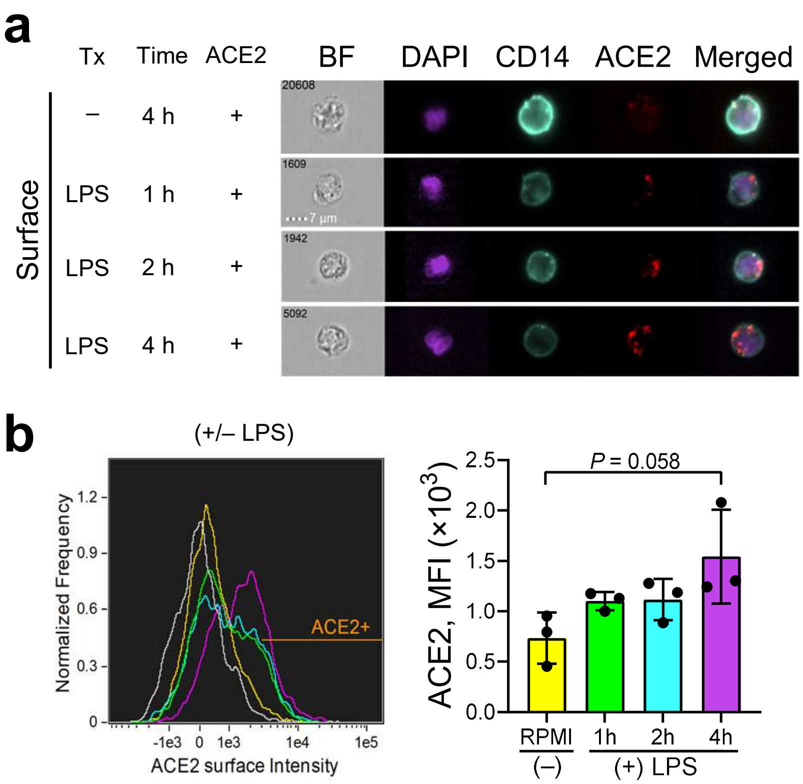

Supplementary Figure S5

Translocation of cytoplasmic ACE2 protein to the cell surface of CD14+ monocytes upon ex vivo stimulation with LPS.

(**a**) Imaging flow cytometry analysis of cytoplasmic and surface ACE2 protein in CD14+ monocytes treated (Tx) with or without LPS up to 4 h. Each cell is represented by the row of images that include bright field (BF), DAPI (purple), CD14 (turquoise), ACE2 (red), and the overlapping image merged with DAPI, CD14, and ACE2. Histograms of ACE2 intensity on the cell surface of CD14+ monocytes treated with LPS (**b**). Grey line represents the isotype control group (Iso). The column charts of mean fluorescence intensity (MFI) of surface ACE2 at other indicated conditions are shown on the right (n = 3 biologically independent samples/group).

Supplementary Figure S6

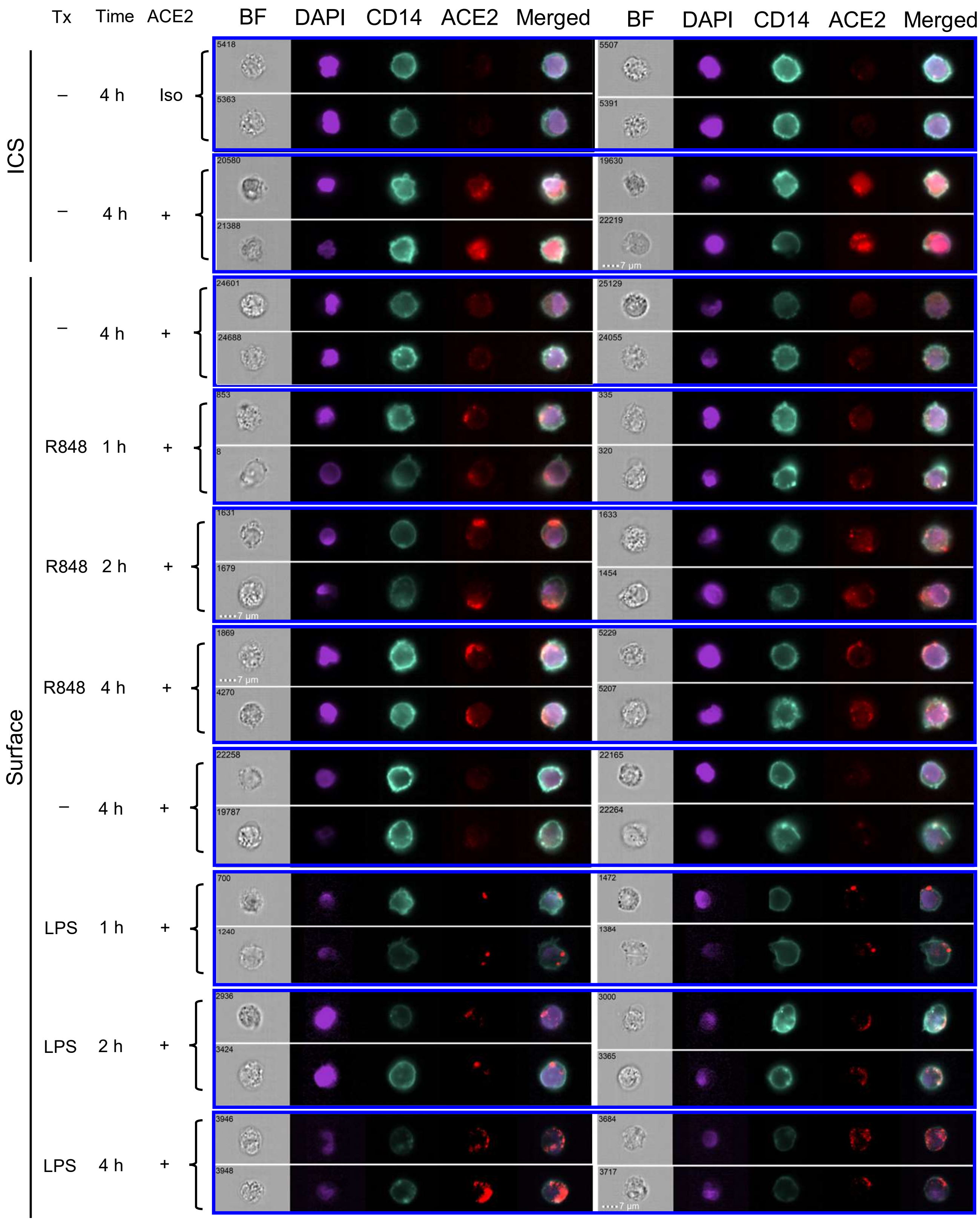

Supplementary Figure S6

Imaging flow cytometry of intracellular and surface ACE2 in peripheral blood monocytes.

PBMCs were treated (Tx) with or without R848 or LPS up to 4 h and analyzed for ACE2 expression on the cell surface or intracellularly (ICS) in $CD14^{+}$ monocytes. Each cell is represented by the row of images that include bright field (BF), DAPI (purple), CD14 (turquoise), ACE2 (red), and the overlapping image merged with DAPI, CD14, and ACE2. Four cells are shown for each condition.

Supplementary Figure S7

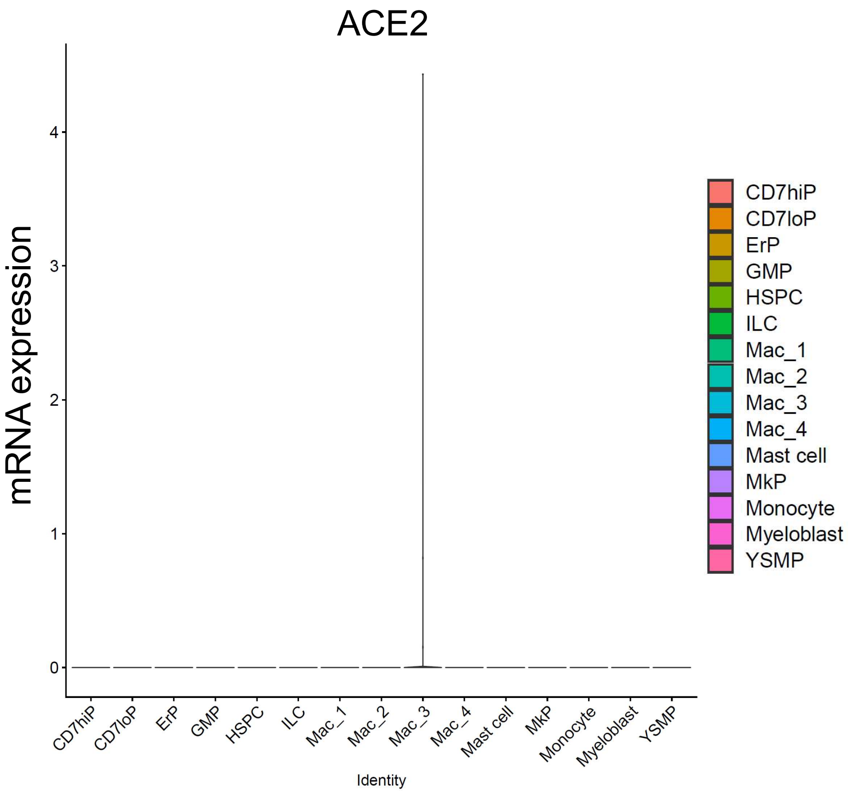

Supplementary Figure S7

ACE2 transcriptomic analysis of CD45$^+$ haematopoietic cells in tissues from human embryos.

CD45$^+$CD235a$^-$ haematopoietic cells were collected from yolk sac, head, liver, blood, skin and lung between Carnegie stage 11-23 (C11-C23) for STRT-seq analysis (n = 8 biologically independent embryo samples). Violin plot showing mRNA expression of ACE2 by 15 identified clusters from the public dataset GSE133345. CD7hi/loP, CD7$^{hi/lo}$ progenitors; ErP, erythroid progenitors; GMP, granulocyte-monocyte progenitors; HSPC, haematopoietic stem and progenitor cells; ILC, innate lymphoid cells; Mac, macrophage; MkP, megakaryocyte progenitors; YSMP, yolk sac-derived myeloid-biased progenitors.

Supplementary Figure S8

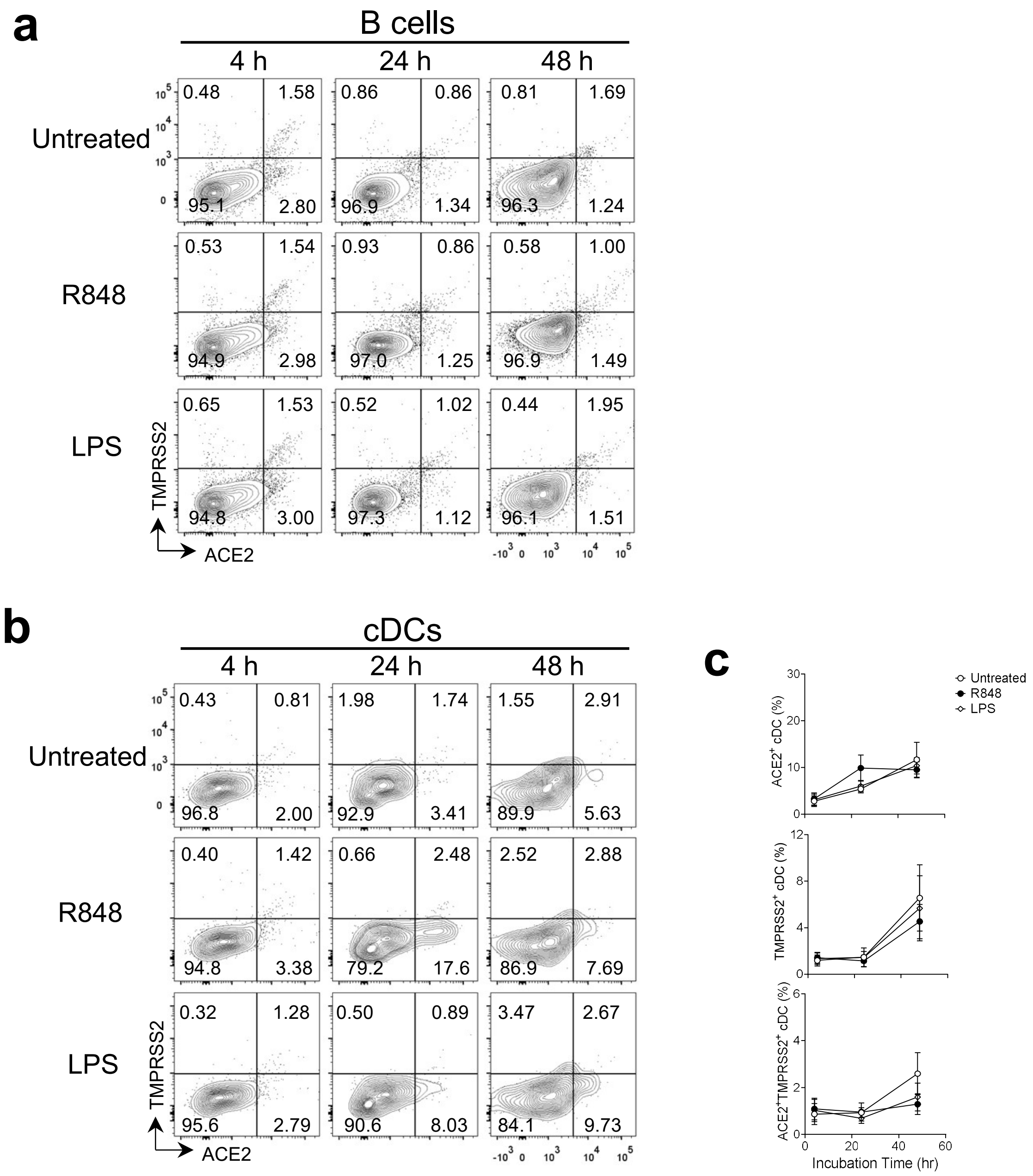

Supplementary Figure S8

Surface expression of ACE2 and TMPRSS2 in B cells and cDC upon exposure to R848 and LPS.

PBMCs were ex vivo cultured with R848, LPS or medium alone for 4 h, 24 h, and 48 h. (**a**) Representative flow cytometric analysis of surface expression of ACE2 and TMPRSS2 in B cells. (**b**) Representative flow cytometric analysis of surface expression of ACE2 and TMPRSS2 in cDC. (**c**) The frequencies of $ACE2^{+}$ cDC, $TMPRSS2^{+}$ cDC and $ACE2^{+}TMPRSS2^{+}$ cDC for each condition in **b**. Bars represent mean ± s.e.m. of biologically independent samples (n = 6/group at each time point).

Supplementary Figure S9

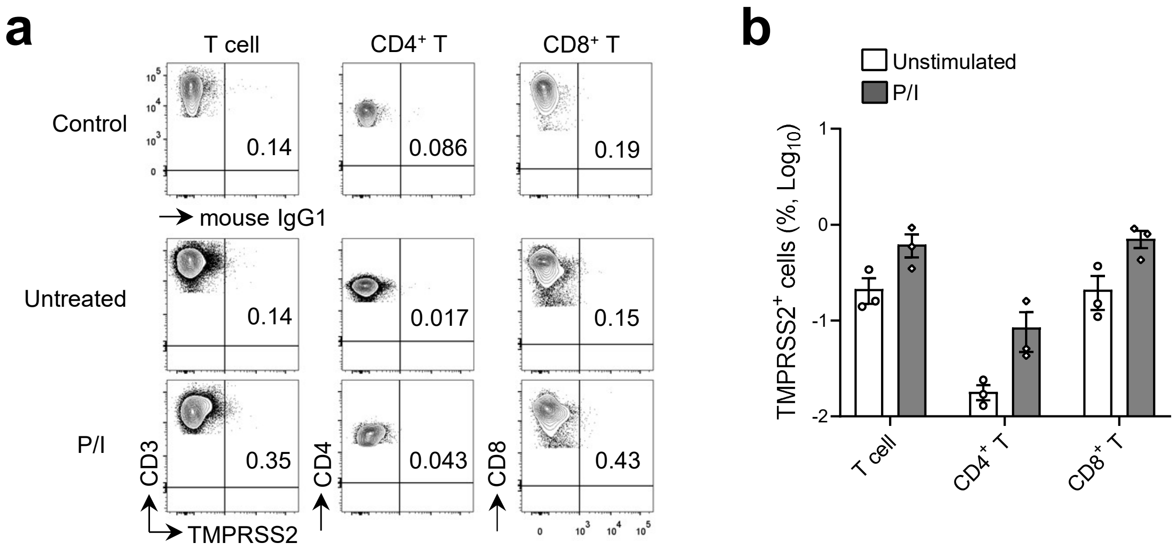

Supplementary Figure S9

Surface expression of TMPRSS2 in circulating T cells upon ex vivo stimulation.

(**a**) PBMCs were ex vivo cultured with PMA plus ionomycin for 4 h. Representative flow cytometric analysis of surface expression of TMPRSS2 in $CD4^+$, $CD8^+$ and total T cells. (**b**) The frequencies of the indicated populations expressing surface TMPRSS2 as in **a**. Bars represent mean ± s.e.m. of biologically independent samples (n = 3/group).

Supplementary Figure S10

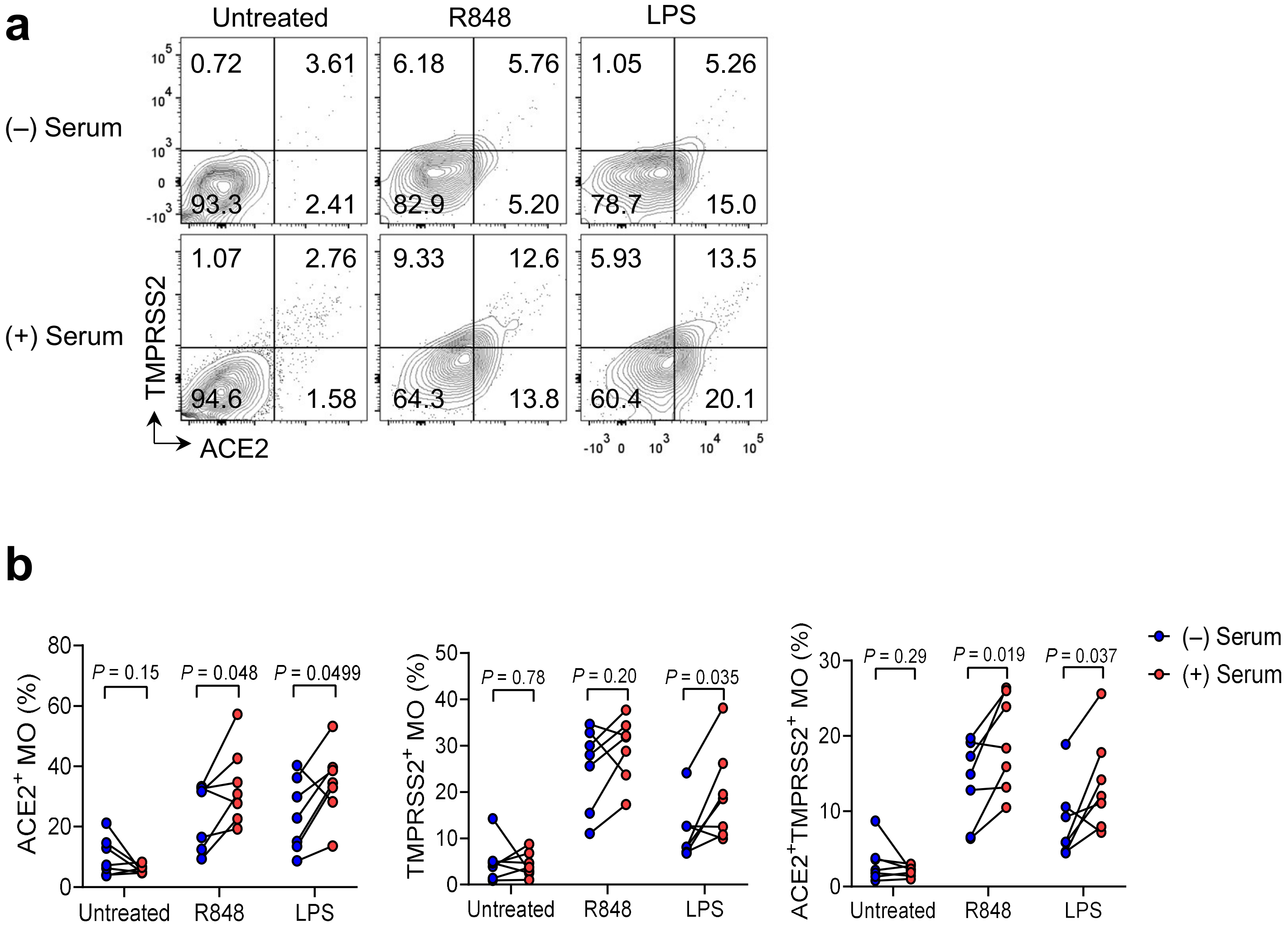

Supplementary Figure S10

Surface expression of ACE2 and TMPRSS2 in monocytes upon exposure to R848 and LPS in the presence or absence of serum.

PBMCs were ex vivo cultured with RPMI1640 medium in the presence or absence of 10% FBS and with R848, LPS, or medium alone for 24 h. (**a**) Representative flow cytometric analysis of surface expression of ACE2 and TMPRSS2 in monocytes (MO). (**b**) The frequencies of ACE2$^+$ MO, TMPRSS2$^+$ MO and ACE2$^+$TMPRSS2$^+$ MO for each condition in **a**. Each dot represents one biologically independent sample (n = 6/group).

Supplementary Figure S11

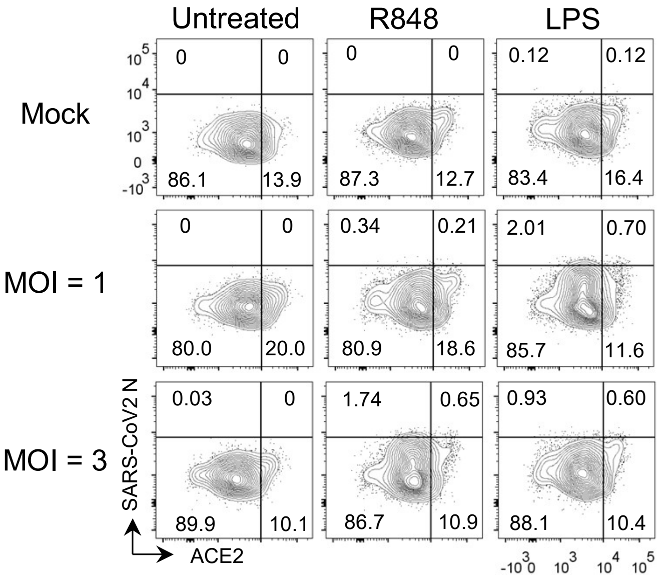

Supplementary Figure S11

cDC are impermissive to SARS-CoV-2 upon exposure to R848 and LPS.

PBMCs were cultured in the presence of R848 or LPS or medium alone for 2 h prior to infection with SARS-CoV-2 at MOI = 1 or 3 for 24 h. Flow cytometry analysis of the surface ACE2 and intracellular SARS-CoV-2 nucleocapsid (N) protein in cDC from mock-infected and virus-infected cells. Data were collected from a pool of PBMCs from 3 healthy control samples. Two independent infection experiments were performed with similar results.

Supplementary Figure S12

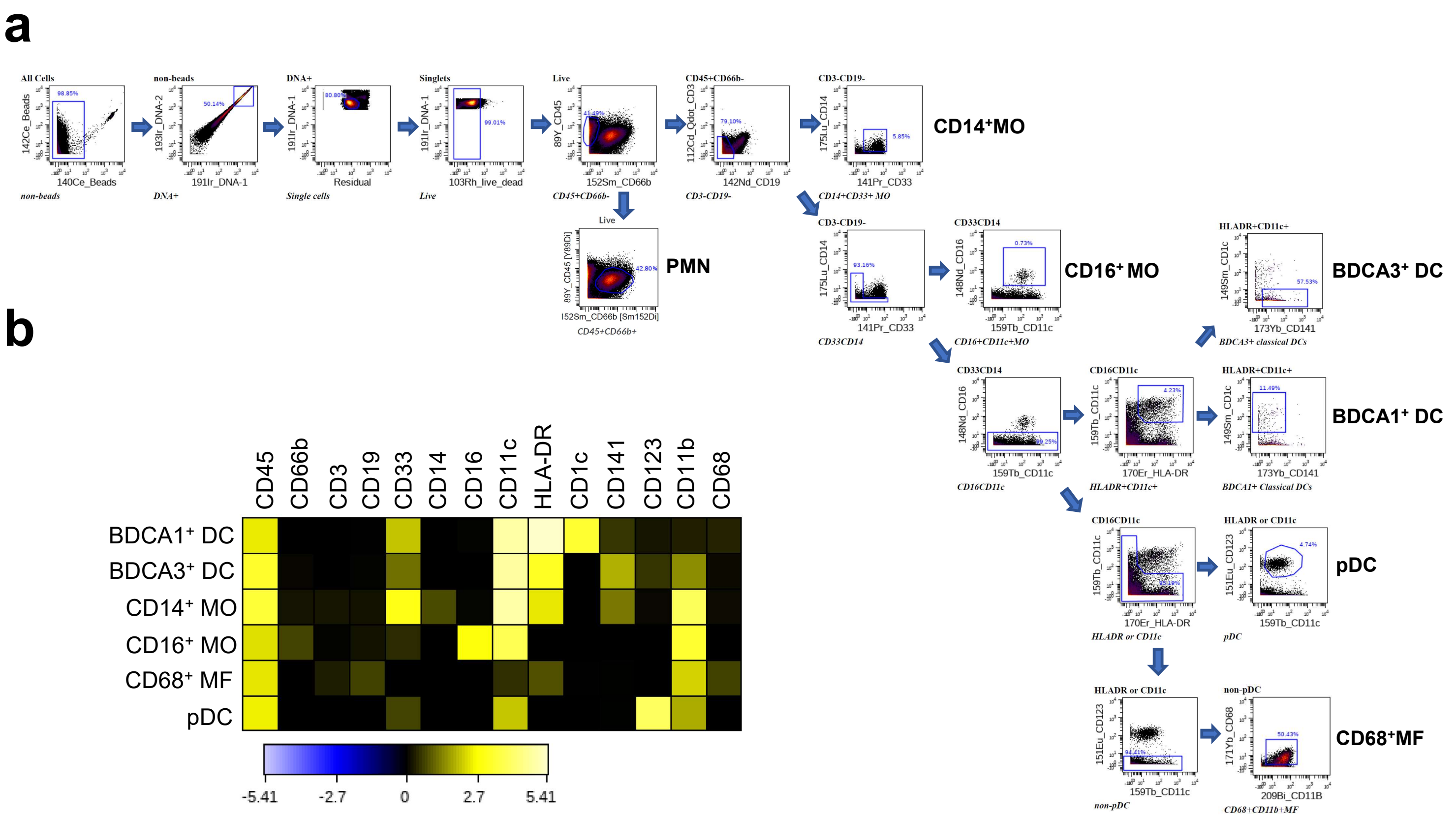

Supplementary Figure S12

CyTOF gating strategy of myeloid populations in whole blood upon ex vivo stimulation.

(**a**) Whole blood samples were stained with 38 metal-conjugated antibodies and analyzed by mass cytometry (CyTOF). After excluding beads, debris, doublets and dead cells, the live singlets were gated based on the expression of CD45 and CD66b. The $CD45^{+}CD66b^{-}$ cells were further gated based on expression of CD3 and CD19. The $CD3^{-}$ $CD19^{-}$ cells were then gated based on the indicated lineage markers to define myeloid populations. Representative data shown are from the blood sample of one moderate COVID-19 patient. (**b**) Heat map showing median intensity of the indicated lineage markers for each myeloid population. The color scale was obtained after calculating transformed ratio of medians by Table's Minimum using values of X-Axis channels in Cytobank platform. MO, monocytes; MF, macrophages.

Supplementary Figure S13

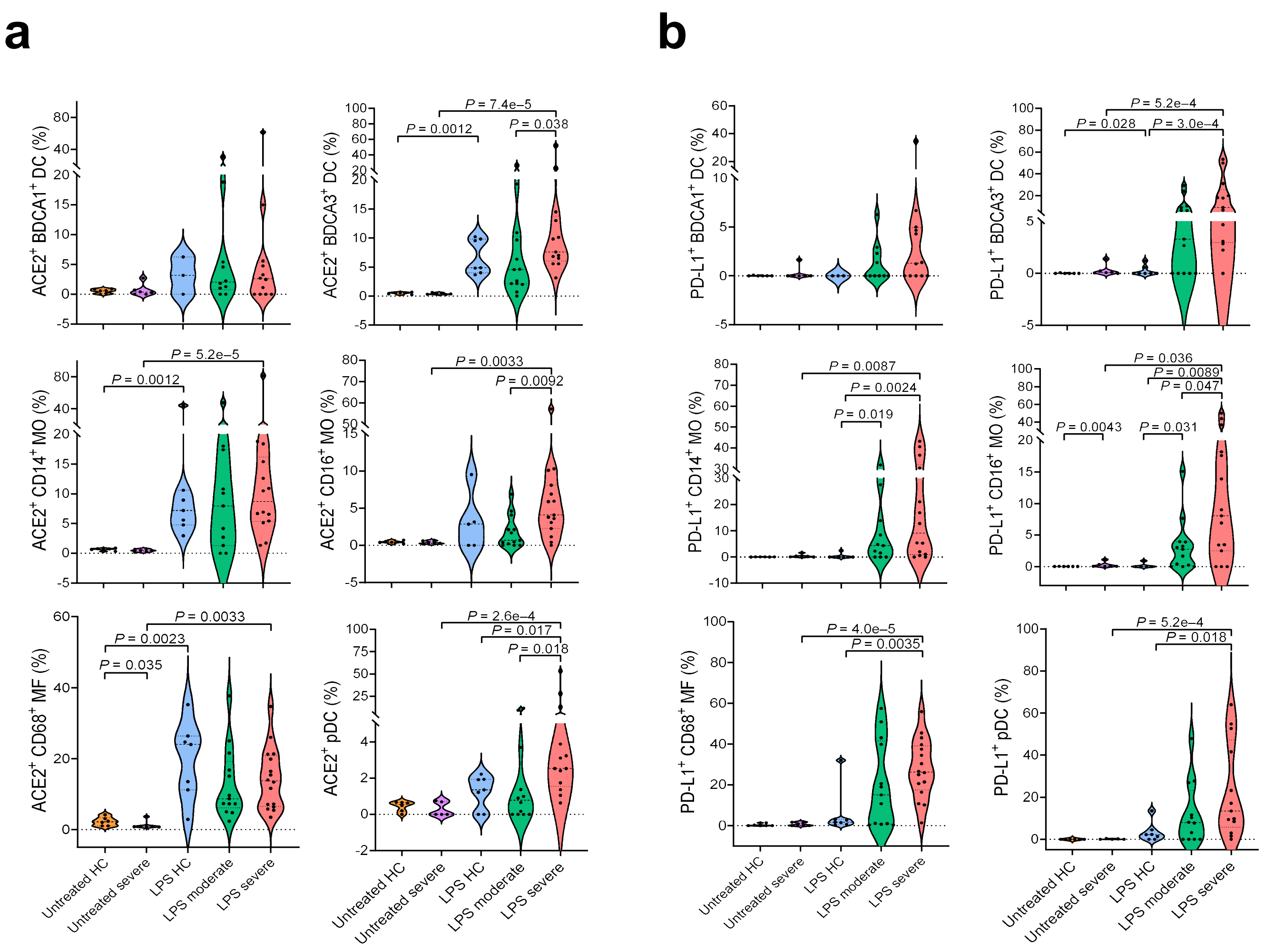

Supplementary Figure S13

Surface expression of ACE2 and PD-L1 in circulating myeloid populations upon LPS ex vivo stimulation.

Whole blood samples were ex vivo stimulated in the presence or absence of LPS for 4 h, and then stained with metal-conjugated antibodies for mass cytometry analysis. Violin plots showing the frequencies of myeloid compartment expressing surface ACE2 (**a**) and PD-L1 (**b**) from untreated samples (n = 7 healthy controls (HC); n = 7 severe COVID-19) as well as LPS-treated samples (n = 7 HC; n = 15 moderate COVID-19; and n = 16 severe COVID-19) of cohort.

Supplementary Figure S14

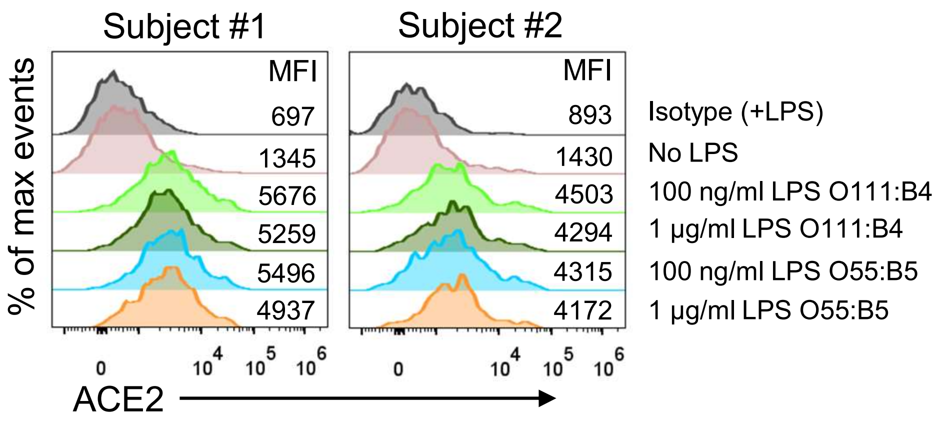

Supplementary Figure S14

Inducible ACE2 surface expression in CD14^{+} monocytes upon LPS ex vivo stimulation.

PBMCs from two healthy subjects were ex vivo stimulated in the presence of LPS O111:B4 or LPS O55:B5 at the indicated concentrations or medium alone for 16 h followed by flow cytometry analysis. Histogram showing ACE2 expression on the cell surface of CD14^{+} monocytes from the treated samples. Mouse IgG1 served as an isotype control. Mean fluorescence Intensity (MFI) of ACE2 shown on the right side of each panel. Experiments were performed independently twice with similar results.

Supplementary Figure S15

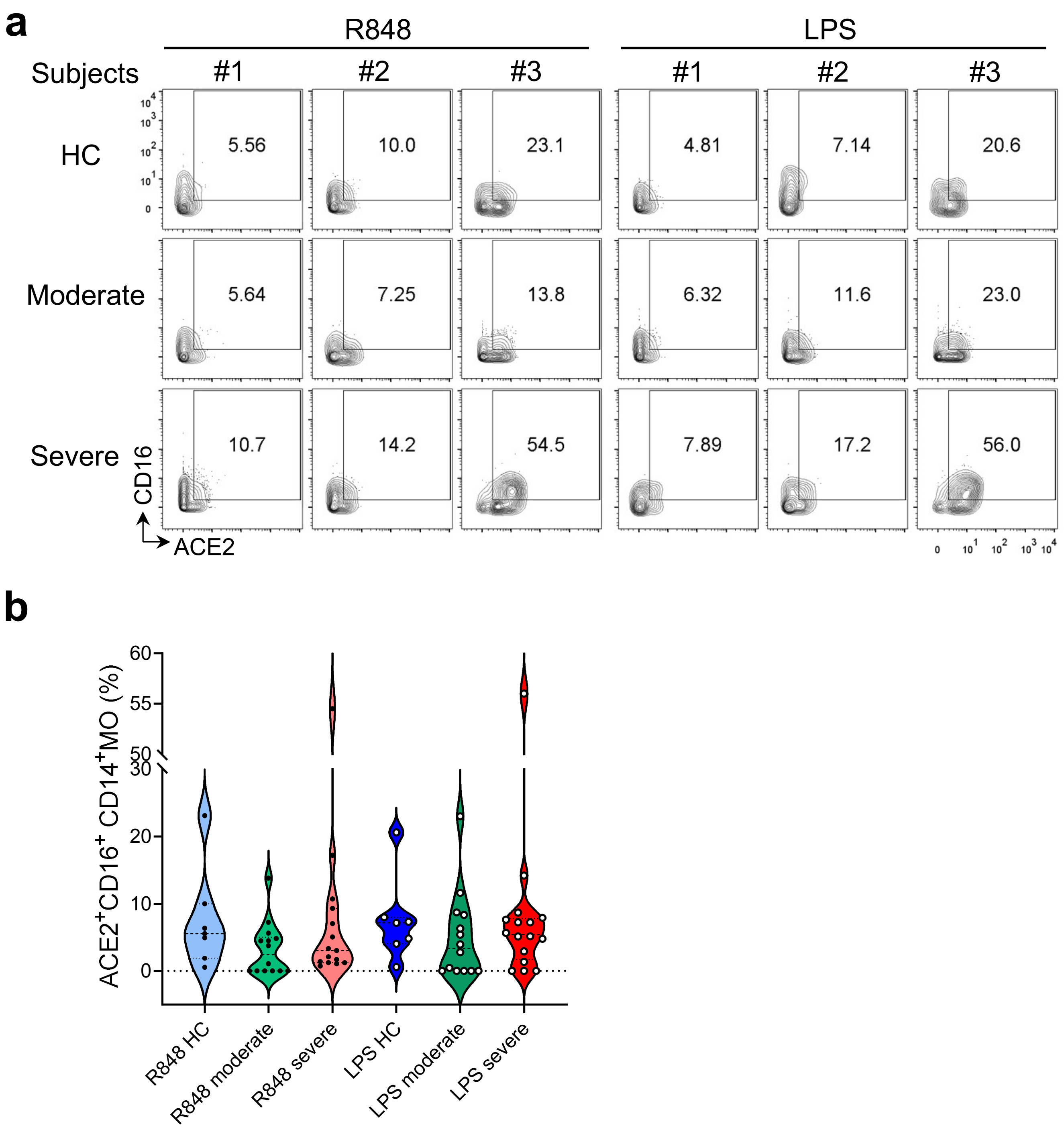

Supplementary Figure S15

Co-expression of ACE2 and CD16 in CD14⁺ monocytes upon R848 or LPS ex vivo stimulation.

Whole blood samples were ex vivo stimulated in the presence or absence of R848 or LPS for 4 h, and then stained with metal-conjugated antibodies for mass cytometry analysis. (**a**) Mass cytometry analysis of ACE2 and CD16 expression on the cell surface of $CD14^+$ monocytes from R848 or LPS-treated (n = 7 HC; n = 14 moderate COVID-19; and n = 15 severe COVID-19) samples. (**b**) Violin plots of the frequencies of $ACE2^+CD16^+$ cells within $CD14^+$ monocytes as shown in (**a**).
